# Sport sustainability research at the intersection of climate action, SDGs, and sport management: a comparative bibliometric mapping of Web of Science and Scopus

**DOI:** 10.3389/frma.2026.1884197

**Published:** 2026-07-03

**Authors:** Fikret Kayhalak, Mehmet Soyal, Tekmil Sezen Soyal, Erhayat Özgur Bayazıtlı, Nuri Muhammet Çelik, Taner Atasoy, Mehmet Sena Kaşka

**Affiliations:** 1 Faculty of Sports Sciences, Istanbul Gelisim University, Istanbul, Türkiye; 2 Faculty of Sports Science, Batman University, Batman, Türkiye; 3 Istanbul Gelisim University Construction Works Department, Istanbul, Türkiye

**Keywords:** bibliometric analysis, climate action, ESG governance, science mapping, Scopus, sport ecology, sport management, sport sustainability

## Abstract

**Purpose:**

This study examines the intellectual, structural, and thematic development of sport sustainability research at the intersection of climate action, Sustainable Development Goals (SDGs), ESG governance, sport ecology, and sport management. It also investigates how Web of Science and Scopus represent this interdisciplinary field and the extent to which database selection influences its visible structure and boundaries.

**Design/methodology/approach:**

Bibliographic data were collected from the Web of Science Core Collection and Scopus covering the period 2010–2025, with the final search conducted on May 11, 2026. Following PRISMA-guided screening, deduplication, and search-field sensitivity analysis, 6,279 unique publications were retained from an initial dataset of 9,711 records. Analyses were performed in R using the bibliometrix package and included performance analysis, Jaccard overlap assessment, database-specific subset comparison, Lotka's and Bradford's laws, keyword co-occurrence analysis, thematic mapping, thematic evolution, and three-fields analysis. The study further interprets bibliometric patterns through Bourdieu's field theory by considering publication concentration, symbolic recognition, and thematic consolidation.

**Findings:**

Sport sustainability research has grown substantially since 2018, with acceleration after 2020. Comparison of Web of Science and Scopus revealed both a shared bibliographic core and a considerable database-specific periphery. Shared records accounted for 54.71% of the final dataset, while WoS-only and Scopus-only records represented 20.10% and 25.20%, respectively. These findings indicate that database selection influences the visible structure, source composition, and thematic coverage of the field. Lotka analysis demonstrated a highly concentrated authorship pattern (β = 2.888, *R*^2^ = 0.975), although a significant K–S deviation reflected the predominance of single-publication authors. Bradford analysis confirmed a clear core–periphery journal structure. Thematic analyses revealed a transition from environmental awareness and event legacy topics toward climate accountability, ESG governance, sport ecology, and SDG-oriented research.

**Research limitations/implications:**

The study is limited to English-language publications indexed in Web of Science and Scopus. Future research should incorporate additional databases, non-English sources, and empirical sustainability indicators to enhance coverage and practical relevance.

**Originality/value:**

By integrating Web of Science and Scopus within a comparative bibliometric framework, this study provides a comprehensive overview of sport sustainability research and demonstrates how database infrastructures influence the representation and development of emerging scientific fields.

## 1 Introduction

Sustainability has become one of the defining concepts of contemporary social, economic, and institutional transformation. Originally associated mainly with environmental protection and resource conservation, the concept has expanded into a multidimensional framework that includes economic resilience, social justice, climate action, governance quality, institutional accountability, and intergenerational responsibility. This broader understanding has been reinforced by the United Nations 2030 Agenda and the Sustainable Development Goals (SDGs), which provide a global framework for addressing interconnected challenges such as climate change, inequality, health, urban sustainability, responsible consumption, and cross-sectoral partnerships.

Within this global transformation, sport has become an increasingly important field of sustainability inquiry. Sport is not merely a recreational or entertainment activity; it is also a large social, cultural, economic, and environmental system. Professional sport organizations, sport events, stadiums, sport tourism, fan mobility, facility management, equipment production, and media-driven sport consumption all generate environmental and social impacts. At the same time, sport has an exceptional capacity to mobilize communities, influence public behavior, communicate social values, promote health, support inclusion, and create collective identities. This dual role has led scholars to conceptualize sport as both part of the sustainability problem and part of the sustainability solution (Hautbois and Desbordes, 2023).

The relationship between sport and sustainability has developed significantly over the past decade. Early research often focused on the environmental impacts of mega-sport events, facility operations, waste management, sport tourism, and event legacy. More recent scholarship has expanded toward climate governance, carbon neutrality, ESG-oriented management, environmental communication, sustainable stadiums, organizational accountability, circular economy, and sport ecology. In this context, (McCullough et al. 2020) conceptualized sport ecology as an emerging subdiscipline within sport management, emphasizing the bidirectional relationship between sport and the natural environment. This perspective is particularly important because climate change is no longer viewed only as an external environmental concern; rather, it is increasingly understood as a structural condition affecting sport participation, athlete health, event scheduling, facility operations, snow and water-based sports, outdoor recreation, and the long-term viability of organized sport (Orr et al., 2022).

The growing visibility of sustainability in sport is also closely related to the SDGs. Sport can contribute directly or indirectly to several SDGs, including SDG 3 (Good Health and Well-Being), SDG 11 (Sustainable Cities and Communities), SDG 12 (Responsible Consumption and Production), SDG 13 (Climate Action), and SDG 17 (Partnerships for the Goals). Sport participation can support physical and mental health; sustainable sport infrastructure can contribute to inclusive and resilient cities; sport organizations can adopt responsible consumption practices; sport events can reduce environmental footprints; and international sport networks can foster multi-stakeholder partnerships. However, the contribution of sport to the SDGs should not be assumed automatically. As (Campillo-Sánchez et al. 2021) argue, sport's contribution to sustainable development requires policy coherence, institutional alignment, shared measurement frameworks, and collaboration among public, private, and civil society actors.

Despite the increasing academic and policy relevance of sport sustainability, the literature remains conceptually broad and structurally fragmented. Environmental sustainability in sport has been reviewed in recent scholarship, revealing growing interest in organizational practices, stakeholder engagement, environmental management, and sustainability implementation (Cury et al., 2023). However, the field continues to span multiple disciplines, including sport management, tourism, public health, urban planning, environmental science, sociology, business, and policy studies. This interdisciplinary structure creates both opportunities and challenges. On the one hand, it enriches the literature by integrating diverse conceptual and methodological perspectives. On the other hand, it complicates the identification of the field's intellectual core, dominant themes, collaboration patterns, and long-term conceptual trajectory.

Bibliometric analysis provides a robust methodological approach for addressing this complexity. By combining performance analysis and science mapping, bibliometric methods make it possible to examine publication growth, source concentration, author productivity, citation impact, collaboration networks, keyword structures, thematic clusters, and thematic evolution within a research field (Donthu et al., 2021). The bibliometrix package developed by (Aria and Cuccurullo 2017) has become one of the most widely used tools for conducting comprehensive bibliometric analysis and science mapping in R. These methods are especially useful for emerging interdisciplinary fields because they allow researchers to identify not only how much a field has grown, but also how its intellectual and conceptual architecture has developed. It is important to acknowledge that bibliometric maps do not provide a neutral mirror of knowledge. Rather, they represent patterns of institutionalized scientific communication shaped by journal gatekeeping, citation conventions, language preferences, publication cultures, and database indexing decisions. In line with the Leiden Manifesto, bibliometric indicators should therefore be interpreted contextually and should not be treated as neutral proxies for scientific quality or knowledge content (Hicks et al., 2015).

Previous bibliometric research has begun to examine sustainability perspectives in sport. In particular, (Boros et al. 2024) provided an important bibliometric overview of sustainability-related scholarship in sport and demonstrated the growing visibility of sustainability perspectives within sport research. However, the present study differs from and extends this antecedent work in several important ways. First, it uses a larger dual-database corpus constructed from both Web of Science and Scopus rather than relying on a single bibliographic source. Second, it explicitly quantifies the degree of overlap and divergence between the two databases through Jaccard similarity analysis. Third, it incorporates search-field sensitivity analysis and deduplication reliability testing to strengthen methodological transparency and reproducibility. Fourth, it examines whether database-specific records contribute only to corpus size or also affect the thematic and structural interpretation of the field. Finally, the study connects bibliometric patterns to field-theoretical mechanisms, interpreting sport sustainability research not only as a growing literature but also as an emerging scientific field shaped by publication hierarchies, symbolic capital, database infrastructures, and thematic boundary formation.

Against this background, database selection represents a major methodological issue in bibliometric research. Web of Science and Scopus are among the most widely used bibliographic databases, but they differ in terms of journal coverage, indexing policies, citation structures, disciplinary representation, and regional scope. (Mongeon and Paul-Hus 2016) demonstrated that these databases do not provide identical representations of scientific fields. (Singh et al. 2021) similarly showed that major bibliographic databases differ substantially in their coverage of journals and disciplines. This issue is particularly important for sport sustainability research because the field is distributed across sport management journals, sustainability journals, environmental science outlets, public health journals, tourism journals, and interdisciplinary publication platforms.

Consequently, bibliometric studies based on a single database may provide only a partial picture of sport sustainability research. A Web of Science-only analysis may emphasize more selective and citation-intensive publication channels, while a Scopus-only analysis may capture broader interdisciplinary diffusion. For a field located at the intersection of climate action, SDGs, and sport management, such database differences may affect not only publication counts but also the visibility of authors, countries, journals, keywords, thematic clusters, and collaboration networks. Therefore, comparing Web of Science and Scopus is not merely a technical decision but a methodological requirement for understanding how database structures shape the representation of sport sustainability knowledge.

### 1.1 Theoretical framing: sport sustainability as an emerging scientific field

This study draws on Bourdieu's theory of the scientific field to interpret sport sustainability research as an emerging and structurally differentiated domain of knowledge production. In Bourdieu's sociology of science, scientific fields are not neutral spaces of knowledge accumulation; they are structured arenas in which scholars, journals, institutions, and countries compete for recognition, legitimacy, and authority through the accumulation of scientific and symbolic capital (Bourdieu, 1975, 1988, 2004). These struggles are mediated through publications, citations, journal prestige, institutional affiliation, and recurrent participation in specialized research conversations.

In this study, bibliometric indicators are not treated as direct measures of scientific quality. Rather, consistent with contextual and critical approaches to research assessment, they are interpreted as traces of the field's structure (Hicks et al., 2015; Waltman and van Eck, 2012; Zupic and Cater, 2015). Author-level indicators such as h-index, g-index, total citations, and publication counts are used to examine the accumulation of scientific capital. Bradford zones are interpreted as publication spaces that contribute to symbolic consecration by distinguishing core, intermediate, and peripheral journals. Lotka's productivity distribution is used to examine whether scientific capital is concentrated among a small recurrent author core or dispersed across a broad occasional author population. Keyword co-occurrence structures are interpreted as indicators of the emerging conceptual doxa of the field, while thematic evolution analysis is used to identify how dominant problem definitions change over time. Finally, the comparison between Web of Science and Scopus is interpreted as a field-boundary mechanism, because database infrastructures influence which publications, journals, authors, and themes become visible within the mapped scientific field.

Therefore, the Bourdieusian framework is operationalized in this study by systematically linking bibliometric indicators to field-theoretical mechanisms. This approach allows the analysis to move beyond descriptive performance reporting and to examine how sport sustainability research is structured through unequal visibility, publication hierarchies, thematic consolidation, and database-mediated boundary formation.

Building on this operational framework, the present study addresses the identified gap by conducting a PRISMA-guided comparative bibliometric analysis of sport sustainability research indexed in Web of Science Core Collection and Scopus between 2010 and 2025. It maps the field's intellectual, structural, and conceptual evolution through performance analysis, database overlap analysis, author and source productivity, Lotka's law, Bradford's law, country collaboration, keyword co-occurrence, thematic mapping, thematic evolution, and three-fields analysis. In addition, the study interprets these indicators through a field-theoretical lens by examining how scientific capital, symbolic recognition, publication hierarchies, thematic consolidation, and database-mediated visibility shape the development of sport sustainability research. In doing so, the study moves beyond a descriptive bibliometric overview and offers a comparative, database-sensitive, and theoretically grounded interpretation of sport sustainability as an emerging scientific field.

The study is guided by the following research questions:

**RQ1**. How has sport sustainability research evolved in terms of annual scientific production and overall growth between 2010 and 2025?
**RQ2**. How do Web of Science and Scopus differ in their coverage and representation of sport sustainability research?
**RQ3**. Which countries, authors, institutions, and sources constitute the main structural and intellectual contributors to the field?
**RQ4**. What are the dominant conceptual clusters and thematic structures in sport sustainability research?
**RQ5**. How has the thematic focus of sport sustainability research evolved across different periods?
**RQ6**. How is sport sustainability research connected to climate action, SDGs, sport ecology, and sport management?
**RQ7**. How do Web of Science-only, Scopus-only, and shared records differ in terms of publication patterns, citation visibility, source distribution, and thematic orientation?
**RQ8**. To what extent do the observed bibliometric structures indicate field consolidation, fragmentation, or unequal accumulation of scientific capital in sport sustainability research?

By answering these questions, this study makes several contributions. First, it provides a comprehensive map of sport sustainability research based on a large merged dataset of 6,279 unique publications. Second, it demonstrates the methodological value of comparing Web of Science and Scopus in an interdisciplinary field by showing how database infrastructures shape not only corpus size but also the visible boundaries of authors, sources, themes, and collaboration structures. Third, it extends previous bibliometric work on sport sustainability by integrating search-field sensitivity analysis, deduplication reliability testing, Jaccard overlap quantification, and database-specific record comparison within a single comparative design. Fourth, it contributes conceptually by operationalizing Bourdieu's field-theoretical concepts through bibliometric indicators and by interpreting sport sustainability research in terms of scientific capital, symbolic recognition, publication hierarchies, thematic consolidation, and database-mediated visibility. Fifth, it clarifies how sport sustainability research has shifted from environmental awareness and event legacy toward climate accountability, ESG governance, sport ecology, and SDG-oriented research. Finally, it identifies future research directions for scholars, sport organizations, policy makers, and sustainability practitioners while also emphasizing that bibliometric evidence should be connected with empirical studies of organizational sustainability performance.

## 2 Method

### 2.1 Research design

This study was designed as a comparative bibliometric and science mapping analysis aimed at examining the intellectual, structural, and conceptual development of sport sustainability research. Bibliometric analysis enables the quantitative examination of scientific production, citation patterns, authorship structures, source productivity, collaboration networks, and conceptual evolution within a defined research field. In this respect, the study combines performance analysis and science mapping procedures to identify not only the volume and distribution of publications but also the knowledge structures underlying the field.

The research design follows a comparative database approach based on Web of Science Core Collection and Scopus. The use of both databases was preferred because bibliometric findings may vary depending on database coverage, indexing policies, journal selection criteria, disciplinary scope, and metadata structures. Previous comparative studies have shown that Web of Science and Scopus differ in terms of journal coverage and disciplinary representation, which may directly affect bibliometric indicators and science mapping results (Mongeon and Paul-Hus, 2016; Singh et al., 2021). Therefore, the present study does not treat database selection as a merely technical decision, but as a methodological component that influences the interpretation of the intellectual and structural architecture of sport sustainability research.

The reporting of the data identification, screening, deduplication, and inclusion stages was organized according to the PRISMA 2020 framework. Although PRISMA was originally developed for systematic reviews and meta-analyses, its transparent reporting logic is widely applicable to bibliometric studies in order to clarify how records were identified, screened, and included in the final corpus (Page et al., 2021). The analytical procedures were conducted in R using bibliometrix, a comprehensive tool developed for science mapping and bibliometric analysis (Aria and Cuccurullo, 2017). Bibliometric performance and science mapping procedures were structured in accordance with widely accepted methodological guidelines for bibliometric research (Donthu et al., 2021).

### 2.2 Population and sample

The population of the study consisted of all scholarly publications indexed in Web of Science Core Collection and Scopus between 2010 and 2025 that addressed sport-related contexts together with sustainability-related concepts. The bibliographic universe was defined through a structured search strategy combining terms related to sport, sport management, sport organizations, sport events, sport tourism, sport ecology, Olympic Games, football, soccer, stadiums, sustainability, sustainable development, climate change, carbon footprint, decarbonization, net-zero transition, ESG, green management, environmental management, circular economy, and Sustainable Development Goals. Table 1 presents the operationalization of the Bourdieusian framework adopted in this study.

**Table 1:** Operationalization of the Bourdieusian framework.

Bourdieusian concept	Bibliometric indicator	Interpretation in this study
Scientific capital	h-index, g-index, total citations, publication counts	Accumulated scholarly visibility and recognition
Symbolic capital	Highly cited authors, Bradford core journals	Consecrated positions within the field
Field position	Bradford zones, author productivity, country productivity	Core, intermediate, and peripheral locations in the field
Illusio	Recurrent publication activity, sustained author productivity	Continued investment in the sport sustainability research field
Doxa	Dominant keyword clusters and central themes	Taken-for-granted conceptual priorities of the field
Field boundary	WoS–Scopus overlap, WoS-only and Scopus-only records	Database-mediated visibility and boundary construction
Field consolidation	Lotka distribution, residual deviations, thematic stability	Degree to which the field is stabilizing or remaining fragmented

**Figure 1:**
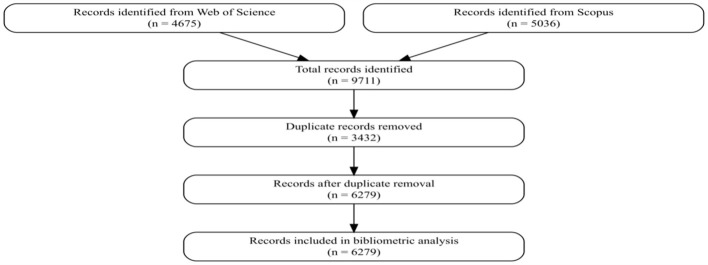
PRISMA flow diagram.

The sample consisted of English-language research articles indexed in Web of Science Core Collection and Scopus that met the predefined inclusion criteria. The search was conducted on May 11, 2026. Initially, 4,675 records were retrieved from Web of Science and 5,036 records from Scopus. Thus, the initial raw dataset consisted of 9,711 records. After DOI- and title-based duplicate removal, 3,432 duplicate records were excluded. The final dataset included 6,279 unique publications for bibliometric analysis. Among these records, 1,262 were unique to Web of Science, 1,582 were unique to Scopus, and 3,435 were indexed in both databases, as shown in Table 2 and Figure 1.

**Table 2:** PRISMA flow summary.

Stage	Description	Records
Identification	Records identified from Web of Science	4,675
Identification	Records identified from Scopus	5,036
Screening	Duplicate records removed using DOI and title-based matching	3,432
Included	Final unique records included in bibliometric analysis	6,279
Included	Records unique to Web of Science	1,262
Included	Records unique to Scopus	1,582
Included	Records indexed in both databases	3,435

Table 2 presents the PRISMA-based record identification, screening, deduplication, and inclusion process used to construct the final bibliometric dataset. The initial search retrieved 4,675 records from Web of Science and 5,036 records from Scopus, resulting in a total of 9,711 raw records before duplicate removal. This initial corpus indicates that sport sustainability research has developed into a sizeable and multidisciplinary literature distributed across both major bibliographic databases.

The screening stage identified and removed 3,432 duplicate records through DOI- and title-based matching. This step is methodologically critical because Web of Science and Scopus partially overlap in their journal coverage and frequently index the same publications. Without systematic deduplication, the bibliometric indicators would have been artificially inflated, particularly in analyses of annual production, author productivity, country output, journal productivity, and keyword frequency.

After duplicate removal, 6,279 unique publications were included in the final bibliometric dataset. The distribution of records also provides important insight into database coverage. Of the final corpus, 1,262 records were unique to Web of Science, 1,582 were unique to Scopus, and 3,435 were indexed in both databases. This pattern indicates that Web of Science and Scopus share a substantial bibliographic core, but they are not interchangeable sources. The presence of a sizeable database-specific periphery also shows that database selection can affect the visible boundaries of sport sustainability research. Therefore, Table 2 supports one of the central methodological arguments of this study: a dual-database strategy provides a broader, more balanced, and more reliable representation of sport sustainability research than a single-database approach.

Figure 1 visually summarizes the PRISMA-based flow of records from database identification to final inclusion. The diagram shows that the dataset was not created through simple aggregation but through a structured and reproducible process involving identification, deduplication, and final eligibility for bibliometric analysis. The two initial sources, Web of Science and Scopus, contributed 9,711 raw records in total, which were subsequently processed through a duplicate removal stage.

The visual structure of Figure 1 makes the reduction from 9,711 raw records to 6,279 unique publications transparent. This transparency is important for bibliometric reproducibility because the construction of the dataset directly affects all subsequent analytical outputs, including performance analysis, Lotka's law, Bradford's law, keyword co-occurrence analysis, thematic mapping, and country collaboration networks. By documenting the data flow clearly, Figure 1 strengthens the methodological credibility of the study.

The figure also highlights the rationale for using PRISMA in a bibliometric context. Although PRISMA was originally developed for systematic reviews, its logic of transparent record identification and screening is useful for bibliometric studies that involve multiple databases and deduplication procedures. In this study, Figure 1 demonstrates that the final corpus of 6,279 unique publications was obtained through a traceable and systematic procedure, thereby enhancing the reliability and interpretability of the subsequent science mapping analyses.

### 2.3 Data sources and search strategy

Bibliographic data were retrieved from Web of Science Core Collection and Scopus. These two databases were selected because they are among the most widely used sources for bibliometric research and provide structured bibliographic metadata suitable for performance analysis, citation analysis, co-authorship analysis, source analysis, and keyword-based science mapping.

The Web of Science search was conducted using the Topic field. The following search query was used:

TS=((sport^*^ OR “sport management” OR “sport organization^*^” OR “sport organisation^*^” OR “sport club^*^” OR “sport event^*^” OR “sport facilit^*^” OR “sport tourism” OR “sport ecology” OR “sport industry” OR “mega sport event^*^” OR “Olympic^*^” OR football OR soccer OR stadium^*^)AND (sustainab^*^ OR “sustainable development” OR “environmental sustainability” OR “climate change” OR “carbon footprint” OR decarbon^*^ OR “carbon neutral^*^” OR “net zero” OR ESG OR “green management” OR “environmental management” OR “circular economy” OR “SDG^*^” OR “sustainable development goal^*^”))

The Web of Science search was limited to the following parameters:

Publication years: 2010–2025 Document type: Article Language: English Index: Web of Science Core Collection

The Scopus search was conducted in the Title, Abstract, and Keywords fields using the following query:

TITLE-ABS-KEY ((sport^*^ OR “sport management” OR “sport organization^*^” OR “sport organisation^*^” OR “sport club^*^” OR “sport event^*^” OR “sport facilit^*^” OR “sport tourism” OR “sport ecology” OR “sport industry” OR “mega sport event^*^” OR “Olympic^*^” OR football OR soccer OR stadium^*^) AND (sustainab^*^ OR “sustainable development” OR “environmental sustainability” OR “climate change” OR “carbon footprint” OR decarbon^*^ OR “carbon neutral^*^” OR “net zero” OR ESG OR “green management” OR “environmental management” OR “circular economy” OR “SDG^*^” OR “sustainable development goal^*^”)) AND PUBYEAR > 2009 AND PUBYEAR < 2026 AND DOCTYPE (ar) AND LANGUAGE (english) The Scopus query was syntactically validated before final execution. In particular, Boolean connectors among all sustainability-related terms were checked to ensure that the query reflected the intended logical structure. The corrected query included the operator OR between sustainab^*^ and “sustainable development”. The final Scopus output was exported only after confirming that the complete query was executable and returned stable record counts.

The search was completed on May 11, 2026. Records were downloaded with full bibliographic information and citation metadata where available. Web of Science records were exported in plain text format, while Scopus records were exported in CSV format.

#### 2.3.1 Search-field sensitivity analysis

Because Web of Science and Scopus do not use fully equivalent searchable metadata fields, a search-field sensitivity analysis was conducted. In Web of Science, the Topic field (TS) searches title, abstract, author keywords, and Keywords Plus fields, whereas Scopus TITLE-ABS-KEY searches title, abstract, and author keywords only. This difference may influence record retrieval and may affect database-level comparisons in interdisciplinary bibliometric studies.

To evaluate the possible effect of this field-scope asymmetry, a parallel Web of Science search was conducted by restricting the search to title, abstract, and author keyword fields. The original WoS Topic-based query retrieved 4,675 records, whereas the restricted WoS title/abstract/author-keyword query retrieved 3,969 records. This corresponds to a reduction of 706 records, or 15.10%, compared with the full TS-based WoS query.

The results indicate that Keywords Plus increased the recall of the WoS search. However, the sensitivity analysis also shows that the majority of WoS records remained retrievable under the restricted field strategy. Therefore, the comparative interpretation of Web of Science and Scopus coverage was retained, while the potential influence of WoS Keywords Plus was explicitly acknowledged. The sensitivity results are reported in Supplementary Table S1.

### 2.4 Inclusion and exclusion criteria

The inclusion and exclusion criteria were defined before data analysis to ensure transparency, reproducibility, and thematic relevance.

#### 2.4.1 Inclusion criteria

Studies were included if they met all of the following criteria:

- The publication was indexed in Web of Science Core Collection or Scopus.
- The publication was published between 2010 and 2025.
- The document type was article.
- The publication language was English.
- The title, abstract, or keywords included at least one sport-related term, such as sport, sport management, sport organization, sport club, sport event, sport facility, sport tourism, sport ecology, sport industry, mega sport event, Olympic, football, soccer, or stadium.
- The title, abstract, or keywords included at least one sustainability-related term, such as sustainability, sustainable development, environmental sustainability, climate change, carbon footprint, decarbonization, carbon neutrality, net zero, ESG, green management, environmental management, circular economy, SDG, or Sustainable Development Goals.
- The publication addressed sport-related sustainability issues in organizational, environmental, social, managerial, policy, tourism, facility, event, or governance contexts.

#### 2.4.2 Exclusion criteria

Studies were excluded if they met any of the following criteria:

- The publication was not an article, such as editorial material, book review, letter, note, proceeding, or conference abstract.
- The publication was not written in English.
- The publication was outside the 2010–2025 publication period.
- The study included sustainability terms but did not include a clear sport-related context.
- The study included sport-related terms but did not address sustainability, climate, environmental management, ESG, SDGs, carbon, circular economy, or related sustainability dimensions.
- Duplicate records appearing in both Web of Science and Scopus were removed using DOI- and title-based matching.
- Records with insufficient bibliographic metadata for bibliometric processing were excluded during data cleaning.

### 2.5 Data collection and PRISMA-based screening process

The data collection and screening process was structured according to the PRISMA 2020 reporting logic. First, bibliographic records were identified through database-specific searches in Web of Science Core Collection and Scopus. In the identification stage, 4,675 records were retrieved from Web of Science and 5,036 records from Scopus. The total number of raw records before deduplication was therefore 9,711.

In the screening stage, records from the two databases were merged and standardized. Duplicate records were identified using DOI-based matching as the primary criterion. For records without DOI information, title-based matching was applied after title normalization. This process resulted in the removal of 3,432 duplicate records. Following deduplication, 6,279 unique records remained in the final dataset. Of these records, 1,262 were indexed only in Web of Science, 1,582 only in Scopus, and 3,435 in both databases. The PRISMA flow summary is presented in Table 2 and the corresponding flow diagram is shown in Figure 1.

This process ensured that the final dataset was transparent, reproducible, and suitable for bibliometric analysis. The use of a dual-database strategy reduced the risk of database-specific coverage bias and enabled a more comprehensive mapping of sport sustainability research.

### 2.6 Data cleaning and harmonization

Data cleaning was conducted before bibliometric analysis to ensure the consistency and reliability of the dataset. Web of Science and Scopus records were first converted into a bibliographic data frame using the convert2df() function in the bibliometrix package. Because the two databases use different metadata structures, the datasets were harmonized into a common bibliometric format.

The cleaning process included several steps. First, DOI fields were standardized by converting all DOI strings to lowercase and removing URL prefixes such as “https://doi.org/” and “doi: ”. Second, titles were standardized by converting text to uppercase, removing extra spaces, and normalizing non-standard characters. Third, source titles, author names, publication years, author keywords, Keywords Plus, and affiliation-related fields were checked for consistency. Fourth, duplicate records were detected through a two-stage deduplication protocol. In the first stage, exact DOI matching was applied after DOI normalization. In the second stage, records without DOI information were evaluated using title-based matching. Titles were standardized by converting all characters to uppercase, removing punctuation, normalizing non-standard characters, deleting extra whitespace, and harmonizing typographic variants. Exact title matching was applied first. For near-duplicate cases, normalized title strings were compared and manually verified when titles showed minor variations due to subtitles, punctuation, spelling differences, or database-specific formatting. Final duplicate decisions were based on combined evidence from title, authorship, publication year, source title, DOI, and document metadata.

DOI matching was prioritized because it provides the most reliable document-level identifier. Title matching was used as a secondary strategy for records without DOI information.

The deduplicated dataset was saved as the master analysis file and used in all subsequent analyses. This procedure produced a final corpus of 6,279 unique publications. The cleaning process was performed in R version 4.6.0 (2026-04-24) using RStudio 2026.04.0.

#### 2.6.1 Deduplication reliability check

To validate the reliability of the deduplication protocol, a random validation sample of 200 records was independently reviewed by two researchers. The reviewers evaluated whether each potentially duplicated record represented the same publication by comparing DOI, title, authorship, publication year, source title, and document metadata. Inter-rater agreement was assessed using Cohen's kappa coefficient, which evaluates the degree of agreement between independent raters beyond chance (Cohen, 1960). The observed agreement was 92.5%, while the expected chance agreement was 59.0%. The resulting Cohen's kappa value was κ = 0.817, indicating almost perfect agreement according to the commonly used interpretation thresholds proposed by (Landis and Koch 1977). Disagreements were resolved through discussion and consensus before the final dataset was locked. These results support the reliability of the DOI- and title-based deduplication protocol used in the study.

### 2.7 Data analysis

The bibliometric analyses were conducted using R version 4.6.0 (2026-04-24) and RStudio 2026.04.0. The main analytical package was bibliometrix, which is specifically designed for comprehensive bibliometric analysis and science mapping in R (Aria and Cuccurullo, 2017). The overall analytical framework followed the principles of performance analysis and science mapping, which are widely used in bibliometric research to evaluate scientific productivity, intellectual influence, collaboration structures, and conceptual development within a research domain (Noyons et al., 1999; Donthu et al., 2021).

The analyses were organized into four main dimensions: descriptive performance analysis, source and author productivity analysis, collaboration and structural analysis, and conceptual/thematic science mapping.

First, descriptive bibliometric analysis was performed to examine the general characteristics of the dataset. This included annual scientific production, annual growth trend, total number of documents, document distribution, citation indicators, source distribution, and database coverage. Descriptive performance indicators are commonly used in bibliometric studies to provide an overview of the volume, growth, and scientific maturity of a research field (Donthu et al., 2021). Annual scientific production was further evaluated using trend statistics. The compound annual growth rate (CAGR) was calculated to quantify the average annual rate of increase in publication output across the study period. In addition, a structural breakpoint analysis was conducted to identify whether the annual production trajectory included a major shift in growth dynamics. These statistics were used to support the interpretation of the annual production trend beyond visual inspection. Structural breakpoints in the annual production trajectory were identified using the Bai–Perron multiple breakpoint test as implemented in the strucchange package in R (Zeileis et al., 2002). This test identifies statistically significant shifts in the mean or trend of a time series and was applied to detect major turning points in annual publication counts.

Second, performance analysis was conducted to identify the most productive and influential countries, authors, institutions, and sources. Author-level productivity was evaluated using publication counts and fractionalized authorship scores. Author-level impact was assessed using h-index, g-index, m-index, total citations, number of publications, and publication start year. These indicators were used to distinguish between simple productivity and sustained scholarly influence. The use of productivity and impact indicators is consistent with the bibliometric performance analysis approach, which aims to evaluate the contribution and influence of scientific actors within a field (Noyons et al., 1999; Donthu et al., 2021).

The bibliographic overlap between Web of Science and Scopus was quantified using the Jaccard similarity coefficient, calculated as the ratio of shared records to the total number of unique records in the merged dataset.

In addition to calculating the Jaccard similarity coefficient, the revised analysis compared three database-defined subsets: Web of Science-only records, Scopus-only records, and records indexed in both databases. These subsets were compared in terms of annual publication distribution, citation visibility, source distribution, and keyword profile. This additional step was conducted to determine whether database-specific records merely increased corpus size or whether they substantively affected the thematic and structural interpretation of sport sustainability research. This comparison also enabled the study to examine database infrastructures as mechanisms that shape the visible boundaries of the scientific field.

Third, Lotka's law was applied to examine the distribution of author productivity. Lotka's law is one of the classical bibliometric laws and assumes that scientific productivity follows an inverse power-law distribution, where a large number of authors publish only one paper while a small number of authors contribute repeatedly to a field (Lotka, 1926). In this study, Lotka's law was used to determine whether sport sustainability research is characterized by a small core of highly productive authors and a large periphery of occasional contributors. The goodness-of-fit of Lotka's law was assessed using the Kolmogorov–Smirnov test, and the estimated Lotka exponent (β), constant (C), coefficient of determination (*R*^2^), K-S statistic (D), and *p*-value were reported.

Because the Kolmogorov–Smirnov test indicated a statistically significant deviation from the theoretical Lotka distribution, a residual inspection was added to the analysis. Observed and expected author frequencies were compared across productivity levels in order to identify whether the deviation was mainly associated with an excess of single-publication authors, a deficit of mid-range contributors, or the disproportionate presence of a small highly productive author core. This step was used to interpret whether sport sustainability research shows signs of field consolidation or remains fragmented around a broad peripheral author population.

Fourth, Bradford's law was used to identify the core publication sources of sport sustainability research. Bradford's law assumes that scientific literature on a given topic tends to concentrate in a limited number of core journals before dispersing across a broader set of peripheral sources (Bradford, 1934). In this study, Bradford's law was applied to determine the source-level concentration structure and to identify the journals that function as core publication outlets in the field. Bradford zones were defined by dividing the ranked source distribution into three zones representing the core, intermediate, and peripheral publication spaces of the field. For each zone, the number of sources was calculated, and Bradford multipliers were estimated by comparing the number of sources across successive zones. This procedure was used to evaluate whether sport sustainability research displays a Bradford-type core–periphery publication structure.

Fifth, country-level productivity and collaboration analyses were conducted to examine the geographical and relational structure of the field. Country productivity was evaluated using publication counts, single-country publications (SCP), multiple-country publications (MCP), and MCP ratio. Country collaboration was visualized through network analysis. Co-authorship and collaboration networks are frequently used in science mapping studies to reveal the social and relational structure of scientific production (Donthu et al., 2021; Aria and Cuccurullo, 2017).

Sixth, keyword co-occurrence analysis was conducted to examine the conceptual structure of sport sustainability research. Co-word analysis is based on the assumption that frequently co-occurring keywords reflect the conceptual and thematic relationships within a research field. This technique is widely used to map the knowledge structure of scientific domains and to identify dominant, emerging, or connected research themes (Callon et al., 1983; Donthu et al., 2021). In the present study, author keywords (DE) were used as the unit of analysis. The minimum frequency threshold was set at three occurrences per keyword. This threshold yielded 1,713 eligible keywords, from which the 250 most frequent keywords were retained for network construction and thematic mapping. Co-occurrence strengths were normalized using the association strength normalization method to reduce the influence of highly frequent terms and to better capture relational proximity among keywords. Thematic mapping was performed using the thematicMap() function in the bibliometrix package with the following parameters: field = “DE”, *n* = 250, minfreq = 3, stemming = FALSE, and repel = TRUE. Thematic clusters were identified through community detection on the weighted keyword co-occurrence network. For each thematic cluster, centrality, density, rank centrality, rank density, and quadrant classification were extracted and reported in Supplementary Table S3. Network-level metrics were calculated for the keyword co-occurrence network in order to assess the structural properties of the conceptual field. The metrics included total number of nodes, network density, clustering coefficient, network diameter, degree centrality, betweenness centrality, and closeness centrality. Degree centrality was used to identify the most connected keywords, betweenness centrality was used to identify bridging concepts between thematic clusters, and closeness centrality was used to assess the relative proximity of keywords within the overall network structure.

Seventh, to evaluate the robustness of the keyword co-occurrence and thematic mapping results, a sensitivity analysis was conducted by varying the minimum keyword frequency and the number of retained terms. The baseline model used minfreq = 3 and *n* = 250. Three alternative models were estimated using minfreq = 5 and *n* = 250, minfreq = 3 and *n* = 100, and minfreq = 5 and *n* = 100. Cluster composition, quadrant classification, and the position of key themes such as climate change, ESG, SDGs, sport ecology, environmental sustainability, and sustainable development were compared across models. Themes that remained in similar positions across models were interpreted as robust, whereas themes that shifted across quadrants were interpreted cautiously as threshold-sensitive. The full thematic mapping sensitivity results are reported in Supplementary Table S7.

Eighth, thematic evolution analysis was conducted to examine how the conceptual structure of sport sustainability research changed across time. The study period was divided into three analytical periods: 2010–2014, 2015–2019, and 2020–2025. Although early-access or metadata-indexed 2026 records appeared in the exported database files due to indexing conventions, these records were excluded from the thematic evolution analysis to avoid incomplete-year indexing bias. The periodization was defined on both conceptual and historical grounds. The first period represents the pre-SDG phase, in which environmental awareness, event legacy, and sport facility issues were more visible. The second period captures the post-2015 SDG institutionalization phase. The third period represents the recent climate-accountability phase, characterized by increased attention to ESG governance, carbon footprint, sport ecology, and SDG-oriented management. The Bai–Perron breakpoint identified in the annual production analysis was interpreted as a production-level acceleration point rather than as a thematic boundary.

Finally, a three-fields plot was used to examine the relationships among countries, keywords, and sources. This analysis enables the simultaneous visualization of how geographical production centers, conceptual orientations, and publication outlets are connected. Three-fields analysis is frequently used in bibliometric studies to reveal the structural relationships among different bibliographic units such as authors, countries, keywords, and journals (Aria and Cuccurullo, 2017).

### 2.8 Ethical considerations

This study used publicly available bibliographic metadata obtained from Web of Science and Scopus. No human participants, personal data, experimental procedures, or intervention-based data were involved. Therefore, ethical approval was not required. The analysis was limited to publication metadata, citation information, source information, author details, keywords, and affiliation data available through bibliographic databases.

### 2.9 Methodological limitations

Several methodological limitations should be acknowledged. First, the study relied only on Web of Science Core Collection and Scopus. Although these databases are widely used in bibliometric research, they do not cover all scholarly outputs, especially books, book chapters, national journals, non-English publications, policy documents, and gray literature. Second, the study included only English-language articles, which may have excluded relevant studies published in other languages. Third, the search strategy was designed to balance sensitivity and specificity; however, because sport sustainability is an interdisciplinary field, some relevant studies may have been missed and some marginally related studies may have been included. Fourth, citation-based indicators are time-sensitive and may favor older publications. Finally, database export structures, indexing practices, and metadata completeness may influence bibliometric results.

In addition, the database-specific subset analysis shows that the visible boundaries of the field remain partly dependent on database coverage decisions. Keyword-based thematic mapping is also sensitive to threshold selection, term retention, and metadata quality, even though sensitivity analyses were conducted to assess robustness. Finally, bibliometric evidence cannot directly measure sustainability practices, ESG implementation, carbon reporting, or organizational transformation in sport organizations; these issues require complementary empirical research.

## 3 Results

### 3.1 Descriptive bibliometric overview

**Figure 2:**
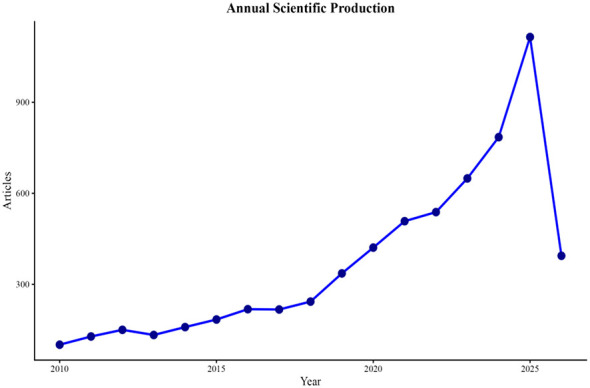
Annual scientific production trend. The year 2026 should be interpreted cautiously because database indexing for that year was incomplete at the time of data retrieval.

Figure 2 shows the annual development trajectory of sport sustainability research. The trend indicates that the field experienced gradual growth during the early years, followed by a more pronounced acceleration after 2018. This pattern suggests that sport sustainability has shifted from a relatively peripheral research topic toward a more visible and institutionally recognized scholarly domain.

The annual trend statistics provide additional support for the growth pattern shown in Figure 2. Scientific production increased from 101 articles in 2010 to 394 articles in 2026, with an estimated annual growth rate of 8.88%. Because 2026 represents an incomplete indexing year, this value was interpreted cautiously and was not treated as evidence of decline. Instead, the overall trajectory indicates sustained growth across the study period. The structural breakpoint analysis identified 2025 as a major turning point, suggesting that the field experienced a pronounced acceleration immediately before the incomplete 2026 indexing window.

This acceleration is consistent with the increasing prominence of climate action, ESG-oriented governance, carbon accountability, sport ecology, and SDG-related research in sport sustainability scholarship. Therefore, Figure 2 does not merely indicate numerical growth; it reflects the broader transformation of sport sustainability into a strategically relevant research area located at the intersection of sport management, environmental policy, public health, and sustainable development.

### 3.2 Database coverage and WoS–Scopus overlap

Table 3 presents the database coverage and overlap structure of the study. A total of 4,675 records were retrieved from Web of Science and 5,036 records from Scopus, resulting in 9,711 raw records before deduplication. After removing duplicate records, the final dataset consisted of 6,279 unique publications. Among these, 3,435 records were indexed in both databases, while 1,262 records were unique to Web of Science and 1,582 were unique to Scopus.

**Table 3:** Database coverage and overlap summary.

Category	Count
Web of Science raw	4,675
Scopus raw	5,036
WoS only	1,262
Scopus only	1,582
Both databases	3,435
Final unique dataset	6,279

These findings show that the two databases have a meaningful overlap, but they are not interchangeable. The presence of 3,435 shared records indicates a common bibliographic core, while the 1,262 WoS-only and 1,582 Scopus-only records demonstrate that each database contributes a substantial database-specific visibility zone. Therefore, Table 3 supports the methodological justification for using a combined database strategy rather than relying on a single bibliographic source.

The degree of bibliographic overlap between Web of Science and Scopus was further quantified using the Jaccard similarity coefficient. The Jaccard coefficient was calculated as the number of records indexed in both databases divided by the total number of unique records in the merged dataset. Based on 3,435 shared records and 6,279 unique records, the Jaccard similarity coefficient was 0.5471, corresponding to an overlap percentage of 54.71%. This value indicates a moderate level of bibliographic overlap. However, it should not be interpreted as evidence that Web of Science and Scopus are interchangeable. Rather, it reveals a dual structure: a shared bibliographic core and a substantial database-specific periphery. Therefore, the database comparison is analytically important not only for increasing corpus size but also for understanding how database infrastructures shape the visible boundaries of sport sustainability research.

#### 3.2.1 Comparative profile of WoS-only, Scopus-only, and shared records

Table 4 presents the comparative profile of WoS-only, Scopus-only, and shared records. The results show that shared records constitute the largest segment of the final dataset, accounting for 54.71% of all publications. This group also displays the highest citation visibility, with a mean of 14.33 citations per record and a median of 6 citations. These findings suggest that records indexed in both Web of Science and Scopus represent the most institutionally visible bibliographic core of sport sustainability research.

**Table 4:** Comparative profile of WoS-only, Scopus-only, and shared records.

Indicator	WoS-only	Scopus-only	Shared records
Number of records	1,262	1,582	3,435
Share of final dataset (%)	20.10	25.20	54.71
Mean publication year	2,022.10	2,019.50	2,021.20
Median publication year	2,024	2,021	2,022
Mean citations per record	9.15	13.93	14.33
Median citations per record	1	3	6
Number of sources	709	896	1,222
Top source	Sustainability	Journal of Physical Education and Sport	Sustainability
Top author keyword	Sustainability	Sustainability	Sustainability
Top 5 author keywords	Sustainability; climate change; physical activity; sustainable development; sport	Sustainability; sustainable development; climate change; sport; sports	Sustainability; climate change; sport; sustainable development; physical activity

The WoS-only subset accounts for 20.10% of the final corpus and has the most recent publication profile, with a mean publication year of 2022.1 and a median publication year of 2024. Its lower citation values, with a mean of 9.15 citations and a median of 1 citation per record, should therefore be interpreted partly in relation to citation-window effects. In other words, WoS-only records appear to capture a more recent segment of the field, which has had less time to accumulate citations.

By contrast, Scopus-only records account for 25.20% of the dataset and show an older publication profile, with a mean publication year of 2019.5 and a median publication year of 2021. This subset also has higher citation visibility than the WoS-only subset, with a mean of 13.93 citations and a median of 3 citations per record. This indicates that Scopus-only records are not merely marginal additions to the corpus; rather, they contribute a substantively visible body of literature that would have been excluded in a Web of Science-only design.

The source and keyword profiles further demonstrate that database-specific records contribute distinct visibility patterns. While *Sustainability* is the leading source in both the WoS-only and shared subsets, the Scopus-only subset is led by *Journal of Physical Education and Sport*. At the author keyword level, “sustainability” is the dominant term across all three subsets, but the top-five keyword profiles differ. WoS-only records emphasize sustainability, climate change, physical activity, sustainable development, and sport; Scopus-only records emphasize sustainability, sustainable development, climate change, sport, and sports; while shared records combine sustainability, climate change, sport, sustainable development, and physical activity.

Taken together, these findings qualify the interpretation of the Jaccard coefficient. The overlap value should not be interpreted only as evidence of a substantial common core. Rather, it indicates a dual structure: a shared bibliographic core and a substantial database-specific periphery. Therefore, the dual-database strategy has substantive interpretive value. It shows that database choice affects not only the size of the corpus but also the apparent citation profile, source structure, temporal distribution, and thematic orientation of sport sustainability research. From a field-theoretical perspective, shared records may be interpreted as the most institutionally visible core of the field, whereas WoS-only and Scopus-only records function as database-specific visibility zones that extend the field's boundaries in different directions.

### 3.3 Country-level scientific production

Table 5 shows the country-level distribution of scientific production in sport sustainability research. China ranks first with 976 publications, followed by the United States with 726 publications and the United Kingdom with 406 publications. These three countries represent the leading national contributors to the field. Australia, Spain, Italy, Germany, and Canada also show substantial productivity, indicating that sport sustainability research is largely concentrated in countries with strong academic infrastructures and established sustainability policy agendas.

**Table 5:** Most productive countries in sport sustainability research.

Country	Articles	Freq	SCP	MCP	MCP_Ratio
China	976	0.1683	151	825	0.845
USA	726	0.1252	101	625	0.861
United Kingdom	406	0.0700	48	358	0.882
Australia	284	0.0490	46	238	0.838
Spain	270	0.0466	30	240	0.889
Italy	250	0.0431	32	218	0.872
Germany	244	0.0421	30	214	0.877
Canada	232	0.0400	35	197	0.849
Korea	167	0.0288	15	152	0.910
India	125	0.0216	44	81	0.648
Turkey	116	0.0200	19	97	0.836
Sweden	99	0.0171	3	96	0.970
Brazil	94	0.0162	16	78	0.830
France	94	0.0162	8	86	0.915
Portugal	92	0.0159	14	78	0.848
Poland	88	0.0152	19	69	0.784
South Africa	83	0.0143	23	60	0.723
Japan	79	0.0136	15	64	0.810
Norway	78	0.0134	9	69	0.885
Switzerland	70	0.0121	8	62	0.886

The MCP ratios in Table 5 provide an important additional layer of interpretation. Countries such as Sweden, France, Korea, Spain, Norway, and Switzerland demonstrate particularly high multiple-country publication ratios. This indicates that sport sustainability research is not only nationally produced but also strongly embedded in international collaboration networks. In contrast, countries with lower MCP ratios may have more nationally bounded research profiles. Overall, Table 5 suggests that the field is shaped by both national research capacity and transnational scientific cooperation.

**Figure 3:**
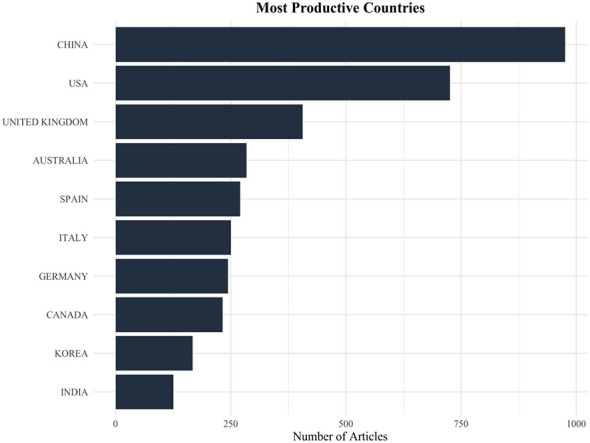
Top 10 countries by scientific production.

Figure 3 visualizes the dominance of the leading countries in sport sustainability research. The figure clearly shows that China, the United States, and the United Kingdom form the primary productivity axis of the field. The gap between these leading countries and the remaining countries suggests that scientific production is not evenly distributed across the global research landscape.

This concentration reflects the role of research funding, institutional capacity, publication infrastructure, and national sustainability agendas in shaping academic productivity. Figure 3 also indicates that sport sustainability research has become particularly visible in countries where sport systems, environmental policy, and higher education research capacity intersect strongly.

**Figure 4:**
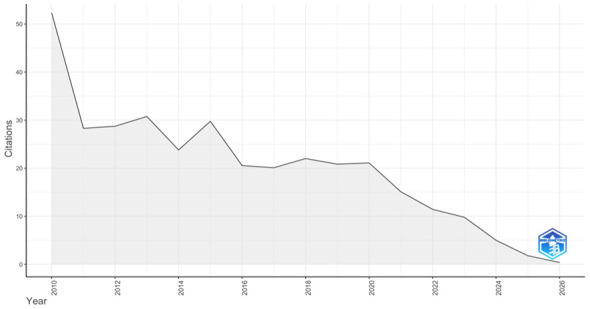
Average total citations per year.

Figure 4 presents the average total citations per year across the study period. The trend shows that earlier publications received higher average citation values, while more recent publications display lower citation averages. This pattern should be interpreted cautiously because citation-based indicators are strongly affected by citation-window effects. Older publications have had more time to accumulate citations, whereas recent publications, particularly those from the incomplete 2026 indexing year, have had limited time to receive citations.

Therefore, Figure 4 should not be interpreted as evidence of declining scientific influence in recent years. Rather, it reflects the time-sensitive nature of citation accumulation in bibliometric analysis. This supports the methodological caution adopted in the study: citation indicators are useful for identifying visibility and accumulated recognition, but they should not be treated as neutral or time-independent measures of scientific quality.

### 3.4 Country collaboration structure

**Figure 5:**
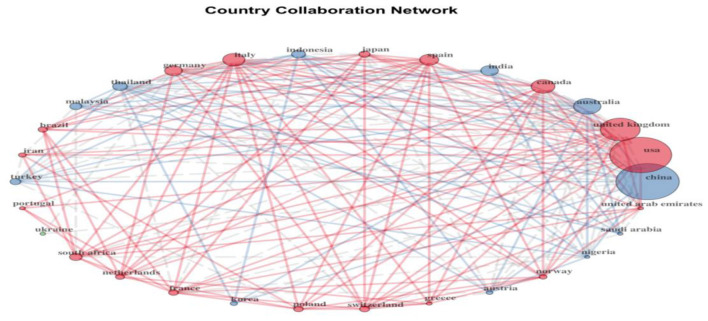
Country collaboration network.

Figure 5 presents the country collaboration network in sport sustainability research. The network structure indicates that the field is characterized by a considerable degree of international connectivity. Countries with high productivity, such as China, the United States, the United Kingdom, Australia, Spain, Germany, Italy, and Canada, also appear to function as important collaboration nodes.

The position of these countries within the network suggests that they do not merely produce a high volume of publications but also contribute to the circulation of knowledge across national boundaries. The networked structure shown in Figure 5 reflects the interdisciplinary and global nature of sport sustainability research. Since sustainability problems in sport involve climate change, event management, infrastructure, mobility, tourism, and governance, international collaboration becomes essential for producing comparative and transferable knowledge.

### 3.5 Author productivity and author-level impact

Table 6 presents the most productive authors in sport sustainability research. McCullough BP ranks first with 37 publications, followed by Zhang Y with 28 publications and Baena-Morales S with 23 publications. Wang Y and Wicker P also appear among the most productive contributors. This distribution indicates that the field has started to develop a recognizable group of leading scholars.

**Table 6:** Most productive authors.

Authors	Articles	Authors	Articles fractionalized
Mccullough BP	37	Mccullough BP	14.18
Zhang Y	28	Wicker P	10.17
Baena-Morales S	23	Zhang Y	8.61
Wang Y	22	Orr M	7.45
Wicker P	22	Ziakas V	6.86
Liu Y	19	Liu Y	6.36
Wang X	19	Baena-Morales S	6.20
Li X	18	Burnett C	6.00
ORR M	18	Wang Y	5.29
Trendafilova S	16	Wang X	5.28
Wang J	16	Koutrou N	5.17
Ferriz-Valero A	14	Trendafilova S	5.02
Kellison T	14	Azzali S	5.00
Zhang X	14	Mallen C	4.75
Li H	13	Wilson B	4.53
Li J	13	Lee J	4.38
Zhang L	13	Kellison T	4.38
Ziakas V	13	Li X	4.37
Kim D	12	Li H	4.35
Kim S	12	Wang J	4.30

The fractionalized authorship values in Table 6 add further nuance to the interpretation. McCullough BP also ranks first in fractionalized productivity, indicating a strong individual contribution beyond participation in collaborative authorship structures. Wicker P, Zhang Y, Orr M, and Ziakas V also show high fractionalized values, suggesting sustained and substantive contributions to the field. Therefore, Table 6 shows that author productivity is not merely a result of large collaborative groups but also reflects consistent individual scholarly engagement.

Table 7 presents the author-level impact indicators based on h-index, g-index, m-index, total citations, number of publications, and starting publication year. McCullough BP has the highest h-index, g-index, total citation count, and m-index among the listed authors. This indicates that McCullough BP combines productivity, citation impact, and continuity, positioning this author as a central intellectual contributor to the field.

**Table 7:** Top authors by h-index.

X	Element	h_index	g_index	m_index	TC	NP	PY_start
1	Mccullough BP	16	32	1.4545455	1035	33	2016
2	Wicker P	11	21	1.2222222	468	22	2018
3	Zhang Y	10	21	1.0000000	456	27	2017
4	Malchrowicz-Mosko E	9	10	1.0000000	208	10	2018
5	Orr M	9	18	1.0000000	448	18	2018
6	Baena-Morales S	8	16	1.3333333	266	23	2021
7	Casper JM	7	7	0.6363636	221	7	2016
8	Daddi T	7	10	1.1666667	165	10	2021
9	Ferriz-Valero A	7	12	1.0000000	160	14	2020
10	González-Serrano MH	7	10	1.0000000	161	10	2020

Wicker P, Zhang Y, Orr M, and Baena-Morales S also demonstrate strong author-level impact. In particular, Baena-Morales S shows a high m-index despite a relatively recent starting year, indicating rapid influence within the field. Table 7 therefore shows that the intellectual structure of sport sustainability research is shaped not only by publication quantity but also by citation visibility and sustained scholarly influence.

### 3.6 Author productivity distribution: Lotka's law

Table 8 presents the author productivity distribution according to Lotka's law. The results show that 16,498 authors produced only one publication, representing 86.28% of all contributing authors. In contrast, 1,728 authors produced two publications, corresponding to 9.04% of the author population. The number of authors declines sharply as the number of publications increases: 474 authors produced three publications, 176 authors produced four publications, and only 82 authors produced five publications.

**Table 8:** Author productivity distribution based on Lotka's law.

Number of publications	Number of authors	Proportion
1	16,498	0.8628
2	1,728	0.0904
3	474	0.0248
4	176	0.0092
5	82	0.0043
6	55	0.0029
7	29	0.0015
8	24	0.0013
9	15	0.0008
10	11	0.0006
11	5	0.0003
12	6	0.0003
13	4	0.0002
14	3	0.0002
16	2	0.0001
18	2	0.0001
19	2	0.0001
22	2	0.0001
23	1	0.0001
28	1	0.0001
37	1	0.0001

This distribution demonstrates a highly asymmetric productivity structure. The field has a broad author base, but most contributors appear only once in the literature. This suggests that sport sustainability research attracts scholars from many adjacent areas, including sport management, tourism, environmental sciences, public health, urban planning, and governance. However, relatively few researchers maintain long-term and repeated publication activity in the field.

The long-tail pattern in Table 8 is particularly important. Only one author reached 37 publications, and very few authors produced more than 20 publications. This indicates that sport sustainability research has a small core of highly productive scholars surrounded by a large peripheral population of occasional contributors. Such a structure is characteristic of interdisciplinary and expanding fields. It suggests that the field has not yet fully consolidated around a narrow set of schools, but it has begun to develop identifiable intellectual leadership.

**Figure 6:**
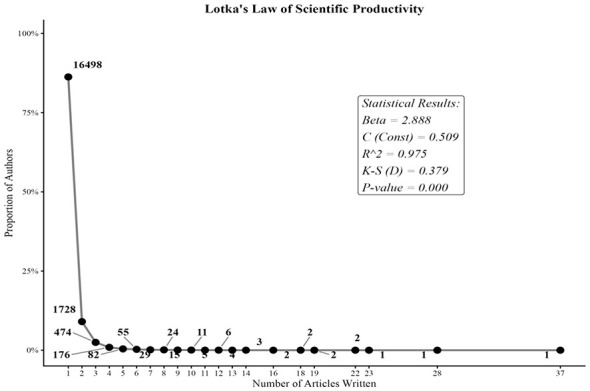
Lotka's law distribution with model statistics.

Figure 6 presents the model-based assessment of author productivity according to Lotka's law. The observed productivity distribution is highly skewed, with the majority of authors contributing only one publication and a very small number of authors producing multiple publications. This pattern confirms the existence of a strong core–periphery structure in sport sustainability research.

The statistical results reported in Figure 6 provide a model-based assessment of this productivity pattern. The estimated Lotka exponent was β = 2.888, the constant was C = 0.509, and the model explained a very high proportion of variance in the observed distribution (*R*^2^ = 0.975). The Kolmogorov–Smirnov test indicated a statistically significant deviation from the theoretical Lotka distribution (D = 0.379, *p* < 0.001). Thus, the observed distribution should not be interpreted as perfect conformity to the theoretical Lotka model. Rather, the high *R*^2^ value indicates a strong Lotka-like power-law structure, suggesting that author productivity in sport sustainability research is highly concentrated among a small number of recurrent contributors.

This finding is important for understanding the intellectual organization of sport sustainability research. The high β value indicates a steeper decline in productivity than the classical inverse-square expectation, suggesting that the field is characterized by a very large population of occasional contributors and a limited number of sustained contributors. In practical terms, sport sustainability research has attracted wide interdisciplinary participation, but long-term productivity remains concentrated among a relatively small group of scholars. This pattern is consistent with an emerging and expanding research field that has begun to develop intellectual leadership but has not yet fully consolidated around a stable and large core author community.

#### 3.6.1 Residual interpretation of Lotka's law

To further examine the significant K-S deviation from the theoretical Lotka distribution, an observed–expected residual analysis was conducted. The analysis compared the observed proportion of authors at each productivity level with the expected proportion derived from the fitted Lotka model. The results indicate that the deviation was not randomly distributed across productivity levels. Rather, it was concentrated primarily at the lowest productivity level. The full observed–expected residual distribution is reported in Supplementary Table S6.

Table 9 presents the observed–expected residual analysis of Lotka's law. The results show that the deviation from the theoretical Lotka distribution is concentrated mainly at the lowest productivity level. The most pronounced difference appears among single-publication authors. While the fitted Lotka model expected 8,266 single-publication authors, the observed number was 16,498. This corresponds to 86.28% of all authors, compared with an expected proportion of 43.23%. The positive residual of 43.05 percentage points indicates a substantial excess of one-time contributors.

**Table 9:** Residual interpretation of Lotka's law by productivity level.

Productivity level	Observed pattern	Residual pattern	Field-theoretical interpretation
Single-publication authors	16,498 authors; 86.28% of all authors	Strong excess compared with expected Lotka proportion	Broad peripheral participation and episodic entry into the field
Low recurrent authors, 2–3 publications	2,202 authors; 11.52% of all authors	Moderate excess compared with expected values	Initial but weak transition from occasional to recurrent contribution
Mid-range contributors, 4–10 publications	392 authors; 2.05% of all authors	Close to expected or slightly deficient at several levels	Underdeveloped stable author community
Highly productive core, >10 publications	Very small number of authors	Sparse but visible recurrent core	Concentrated scientific capital among few actors

Authors with two and three publications also exceeded the expected values, but the residuals were much smaller. Two-publication authors represented 9.04% of the author population, compared with an expected 6.25%, while three-publication authors represented 2.48%, compared with an expected 2.02%. However, from four publications onward, the residual values became very small and, in some cases, slightly negative. For example, authors with five, seven, nine, and ten publications were slightly below the expected proportions.

These results clarify the source of the significant K-S deviation reported in the Lotka analysis. The deviation is driven primarily by the very high proportion of single-publication authors rather than by a large stable group of recurrent contributors. This indicates that sport sustainability research has attracted broad interdisciplinary participation, but much of this participation remains episodic. The field has a very large peripheral author base, while the transition from occasional contribution to sustained authorship appears relatively weak.

From a Bourdieusian field-theoretical perspective, this pattern suggests partial field consolidation rather than full institutional stabilization. A small group of recurrent authors has begun to accumulate scientific capital through repeated publication activity, but the majority of contributors remain positioned at the periphery of the field. Therefore, the residual structure supports the interpretation that sport sustainability research is expanding rapidly while still lacking a broad and stable mid-level author community.

### 3.7 Source-level structure and Bradford's law

Table 10 presents the most productive publication sources in sport sustainability research. Sustainability ranks first with 467 publications, indicating that broad sustainability-oriented journals play a central role in the dissemination of sport sustainability studies. This dominance suggests that the field is not confined to traditional sport science or sport management journals; rather, it is strongly embedded within the broader sustainability science literature.

**Table 10:** Most productive sources.

Sources	Articles
Sustainability	467
Frontiers in Sports and Active Living	102
Sport in Society	74
PLoS ONE	68
Managing Sport and Leisure	62
International Journal of Environmental Research and Public Health	55
Sport Management Review	49
Frontiers in Psychology	48
European Sport Management Quarterly	46
Journal of Cleaner Production	43
International Journal of Sport Policy and Politics	39
Scientific Reports	38
Sport Business and Management-An International Journal	37
Frontiers in Public Health	35
International Journal of Sports Marketing and Sponsorship	34
Journal of Physical Education and Sport	34
International Review for the Sociology of Sport	32
Buildings	30
Journal of Sport Management	30
Journal of Sport and Tourism	29

The presence of journals such as Frontiers in Sports and Active Living, Sport in Society, Managing Sport and Leisure, Sport Management Review, European Sport Management Quarterly, Journal of Sport Management, and International Journal of Sport Policy and Politics shows that sport-specific publication outlets also represent an important part of the field's intellectual infrastructure. At the same time, journals such as PLOS ONE, International Journal of Environmental Research and Public Health, Journal of Cleaner Production, Scientific Reports, Frontiers in Psychology, and Buildings demonstrate the interdisciplinary spread of the topic.

Overall, Table 10 indicates that sport sustainability research is characterized by a dual publication structure. One axis is rooted in sport management, sport policy, sport sociology, and leisure studies, while the other is connected to sustainability science, environmental management, public health, psychology, and built environment research. This duality reflects the hybrid nature of sport sustainability as a research domain that connects organizational, environmental, social, and policy-oriented questions.

Table 11 summarizes the Bradford zone structure of sport sustainability research. The ranked source distribution was divided into three zones representing the core, intermediate, and peripheral publication spaces of the field. Zone 1 consisted of 65 core sources, Zone 2 included 460 intermediate sources, and Zone 3 included 1,816 peripheral sources. This distribution demonstrates that the literature is organized around a relatively limited set of core publication outlets, followed by a substantially broader intermediate and peripheral dissemination space.

**Table 11:** Bradford zone summary.

Bradford zone	Source type	Number of sources	Bradford multiplier	Interpretation
Core/zone 1	Core sources	65	Reference	Central publication outlets of the field
Zone 2	Intermediate sources	460	7.08	Secondary dissemination zone
Zone 3	Peripheral sources	1,816	3.95	Broad interdisciplinary diffusion zone

The Bradford multiplier between Zone 2 and Zone 1 was approximately 7.08, while the multiplier between Zone 3 and Zone 2 was approximately 3.95. Although the observed distribution does not perfectly reproduce the idealized 1:n:n^2^ Bradford pattern, it clearly demonstrates a Bradford-type core–periphery publication structure. This indicates that sport sustainability research has developed identifiable publication centers while also maintaining broad interdisciplinary diffusion across numerous journals. In this respect, the field appears to be simultaneously institutionalizing around a core group of publication outlets and expanding across adjacent domains such as sport management, sustainability science, public health, environmental management, tourism, psychology, and the built environment.

**Figure 7:**
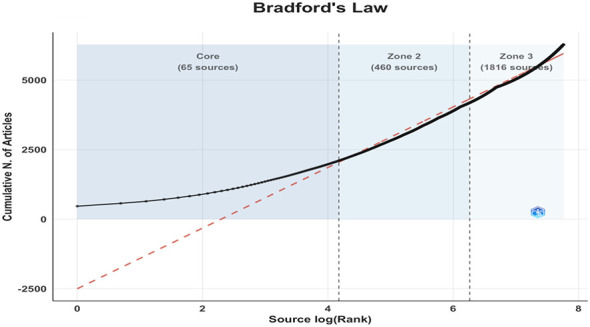
Bradford's law distribution of sources.

Figure 7 visualizes the Bradford distribution of publication sources. The figure confirms that publication output is highly concentrated in a small number of journals, followed by a rapid dispersion into a broader set of less frequently used sources. This pattern is characteristic of a field with an identifiable core literature and a wide interdisciplinary periphery.

The curve presented in Figure 7 demonstrates that sport sustainability research has already developed recognizable publication centers. However, the dispersion beyond the leading journals suggests that the field continues to expand across multiple disciplines. This is important because it indicates that the field is simultaneously consolidating and diversifying: a core set of journals provides stability, while peripheral journals contribute thematic breadth and interdisciplinary reach.

From a knowledge-structure perspective, Figure 7 suggests that sport sustainability research is not fragmented randomly. Instead, its publication pattern follows a structured distribution in which core journals act as primary knowledge repositories. This strengthens the interpretation that the field has achieved a certain level of institutionalization while maintaining openness to interdisciplinary expansion.

### 3.8 Institutional productivity

**Figure 8:**
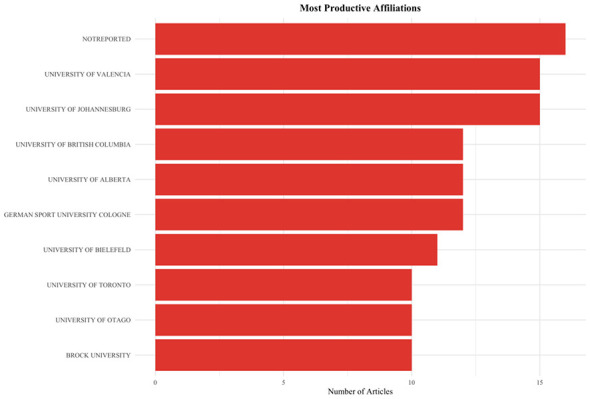
Top 10 affiliations by scientific production.

Figure 8 presents the leading institutional affiliations in sport sustainability research. Institutional productivity is a critical indicator because it reflects not only individual scholarly activity but also the presence of research groups, academic programs, laboratories, interdisciplinary centers, and institutional strategies that support sustained knowledge production.

The concentration of output among the top affiliations suggests that sport sustainability research is increasingly anchored in institutions with strong research infrastructure. These institutions likely provide the academic conditions necessary for interdisciplinary collaboration across sport management, environmental sciences, public health, tourism, urban studies, and sustainability policy. Therefore, Figure 8 indicates that the development of the field is shaped not only by individual authors but also by institutional ecosystems that enable long-term research agendas.

At the same time, the presence of a limited number of dominant affiliations suggests that institutional participation remains uneven. This may create regional and epistemic imbalances in the literature, particularly if institutions from climate-vulnerable or resource-constrained regions remain underrepresented. Future research would benefit from more inclusive institutional collaboration, especially involving universities and research centers from regions where sport sustainability challenges are locally urgent.

### 3.9 Conceptual structure and keyword co-occurrence

**Figure 9:**
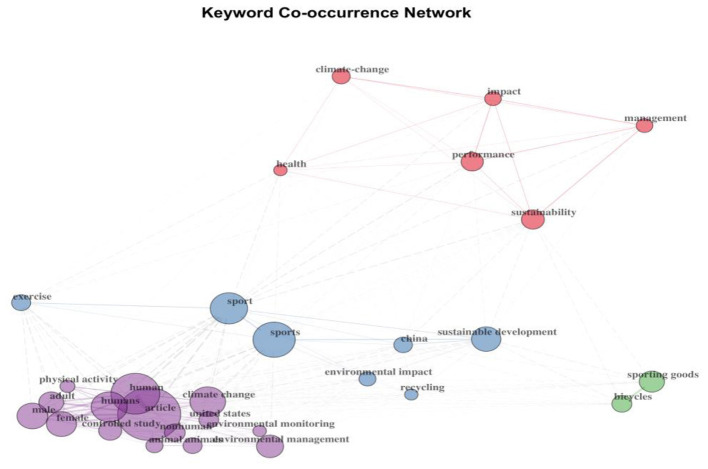
Keyword co-occurrence network.

Figure 9 presents the keyword co-occurrence network and reveals the conceptual organization of sport sustainability research. The network contained 17,523 keyword nodes, indicating a highly extensive and diverse conceptual vocabulary. However, the network density was very low (0.0005), suggesting that although the field includes a large number of keywords, only a limited proportion of all possible keyword connections are actually present. This pattern is typical of broad interdisciplinary research areas in which multiple subfields coexist without being fully integrated into a single dense conceptual structure.

The clustering coefficient was 0.0933 and the network diameter was 11. These values indicate that the keyword network contains identifiable local clusters but remains globally dispersed. In other words, sport sustainability research is not organized around a single compact conceptual core; rather, it consists of multiple thematic regions connected through a limited number of highly central bridging terms.

The centrality results provide a more precise understanding of this structure. Sustainability was the most connected and most influential keyword in the network, with the highest degree centrality (1,900) and betweenness centrality (0.1755). This indicates that sustainability functions as the primary organizing and bridging concept in the field. Climate change was the second most central keyword, with a degree centrality of 1,031 and betweenness centrality of 0.0952, followed by sustainable development, with a degree centrality of 924 and betweenness centrality of 0.0822. These results show that climate action and sustainable development are not peripheral concerns but central conceptual pillars of sport sustainability research.

Other highly connected terms included sport, physical activity, sports, football, tourism, environment, Olympic Games, COVID-19, environmental sustainability, China, physical education, health, sport tourism, sports tourism, exercise, sustainable tourism, and mega-events. This distribution demonstrates that the conceptual structure of the field is organized around the intersection of sport management, environmental sustainability, public health, tourism, mega-events, and climate-related governance. Overall, the keyword network metrics confirm that sport sustainability research has a broad and heterogeneous conceptual structure, but one that is increasingly organized around sustainability, climate change, and sustainable development as central bridging concepts.

**Figure 10:**
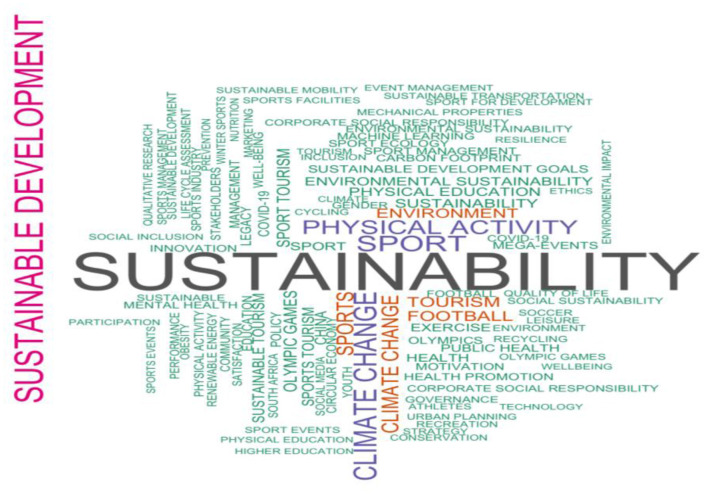
Word cloud of dominant research terms.

Figure 10 visualizes the most dominant research terms in the dataset. The prominence of terms such as sustainability, climate change, sport, sustainable development, environment, football, tourism, Olympic Games, physical activity, and corporate social responsibility indicates that sport sustainability research is built around both environmental and organizational concerns.

The visibility of terms related to climate change and environmental sustainability suggests that ecological issues have become one of the central pillars of the field. At the same time, the presence of sport tourism, football, Olympic Games, and corporate social responsibility shows that sport sustainability is also strongly connected to event management, institutional responsibility, and the social visibility of sport organizations.

Figure 10 therefore supports the interpretation that sport sustainability research has expanded from a primarily environmental discussion into a broader interdisciplinary research domain. The word cloud also reveals that the field combines macro-level global sustainability agendas with meso-level organizational practices and sport-specific contexts.

### 3.10 Thematic structure of sport sustainability research

The numerical centrality and density values of the thematic clusters are reported in Supplementary Table S3. The thematic map identified seven major clusters: sustainability, sport, physical activity, sustainable development, tourism, environmental sustainability, and climate change. These clusters were classified according to their relative position in the centrality–density plane as motor themes, basic themes, niche themes, and emerging/declining themes.

The sustainability cluster had the highest centrality score (0.113), indicating that it functions as the most connected and structurally central theme in the field. However, its moderate density value (3.538) positioned it as a basic theme rather than a fully developed motor theme. This suggests that sustainability operates as a broad conceptual foundation connecting multiple research streams, but its internal thematic development remains distributed across different subdomains.

The sport and physical activity clusters were classified as motor themes. Sport showed high centrality (0.101) and moderate-to-high density (3.604), while physical activity showed strong density (4.151) and substantial centrality (0.085). These findings indicate that sport and physical activity are both well-developed and structurally important themes within the sport sustainability literature. Their motor-theme position suggests that they play an active role in organizing the conceptual development of the field.

Sustainable development was classified as a basic theme, with moderate centrality (0.085) and lower density (2.914). This indicates that sustainable development is central to the field's conceptual structure but remains internally less developed than the motor themes. Tourism was positioned as an emerging/declining theme, with relatively low centrality and the lowest density score. This suggests that sport tourism and sustainability-related tourism research may represent a developing but not yet fully consolidated research trajectory.

Environmental sustainability and climate change were classified as niche themes. Both clusters had high density values, particularly environmental sustainability (4.340) and climate change (4.146), but lower centrality scores. This pattern indicates that these themes are internally well developed but less strongly integrated into the broader conceptual structure of sport sustainability research. Therefore, although climate change and environmental sustainability are theoretically important, the results suggest that they remain specialized clusters rather than fully integrated field-organizing themes.

**Figure 11:**
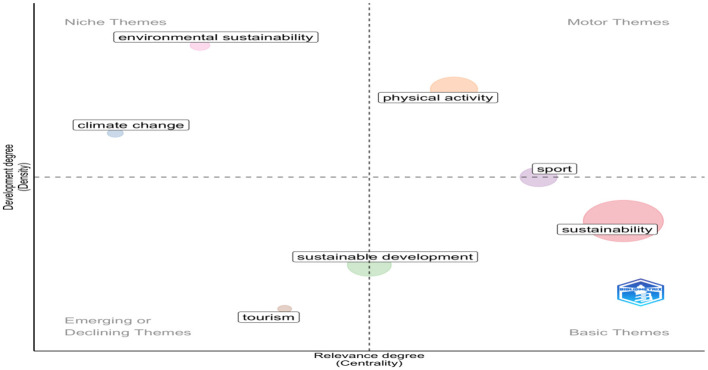
Thematic map of sport sustainability research.

Figure 11 presents the thematic map of sport sustainability research and positions the major themes according to their centrality and density. This structure allows the identification of motor themes, basic themes, emerging or declining themes, and niche themes. Themes with high centrality and high density can be interpreted as highly developed and strongly connected research areas, whereas themes with lower centrality or density may indicate emerging or specialized lines of inquiry.

The thematic map identifies sustainability (centrality = 0.113) and sport (centrality = 0.101) as the most central themes in the field, positioned as basic and motor themes respectively. Physical activity (centrality = 0.085) and sustainable development (centrality = 0.085) occupy strong positions in the motor and basic theme quadrants. Environmental sustainability (centrality = 0.069) and climate change (centrality = 0.032) are classified as niche themes, reflecting high internal density but lower integration into the broader conceptual structure. These data-driven classifications confirm that sport sustainability research includes both well-developed organizing themes and specialized clusters that remain partially peripheral to the field's conceptual core.

Figure 11 also indicates that sport sustainability research is undergoing conceptual consolidation. The coexistence of central and peripheral themes suggests that the field has developed a recognizable thematic structure while still maintaining openness to emerging topics. This is particularly important for a rapidly evolving field shaped by climate politics, ESG frameworks, sport ecology, sustainable facilities, event legacy, and social responsibility.

#### 3.10.1 Sensitivity analysis of thematic mapping

To assess the robustness of the thematic map, a sensitivity analysis was conducted by varying the minimum keyword frequency and the number of retained terms. The baseline model used minfreq = 3 and *n* = 250. Three alternative models were tested: Model A used minfreq = 5 and *n* = 250, Model B used minfreq = 3 and *n* = 100, and Model C used minfreq = 5 and *n* = 100.

Table 12 reports the sensitivity analysis of thematic mapping across four alternative specifications. The baseline model used minfreq = 3 and *n* = 250, while the alternative models varied the minimum keyword frequency and the number of retained terms. The results show that some thematic classifications were stable across specifications, whereas others were sensitive to threshold selection.

**Table 12:** Sensitivity analysis of thematic mapping across alternative thresholds.

Core concept	Baseline (minfreq = 3, *n =* 250)	Model A (minfreq = 5, *n =* 250)	Model B (minfreq = 3, *n =* 100)	Model C (minfreq = 5, *n =* 100)
Climate change	Niche themes	Emerging/declining	Niche themes	Emerging/declining
Environmental sustainability	Niche Themes	Niche themes	Motor themes	Motor themes
Sustainability	Basic themes	Motor themes	Motor themes	Motor themes
Sport	Motor themes	Motor themes	Motor themes	Motor themes
Physical activity	Motor themes	Motor themes	Motor themes	Motor themes

The most stable themes were “sport” and “physical activity,” both of which remained classified as motor themes across all four models. This indicates that these themes occupy structurally important and well-developed positions within the keyword co-occurrence network, regardless of the threshold configuration. Their stability suggests that sport- and physical-activity-related research functions as a central conceptual axis within the broader sport sustainability literature.

By contrast, “climate change” showed clear threshold sensitivity. It was classified as a niche theme in the baseline model and Model B, but shifted to an emerging/declining theme in Model A and Model C. This finding indicates that the position of climate change within the thematic map depends on the retained keyword structure and frequency threshold. Therefore, the classification of climate change as a niche theme should be interpreted cautiously. Rather than treating it as a fixed thematic position, the results suggest that climate change represents a developing and structurally unstable theme within sport sustainability research.

“Environmental sustainability” also showed threshold sensitivity. It appeared as a niche theme in the baseline model and Model A but shifted to a motor theme in Models B and C. This indicates that environmental sustainability becomes more central and developed when the retained thematic structure is narrowed. Similarly, “sustainability” moved from a basic theme in the baseline model to a motor theme in the alternative models, suggesting that its thematic role is robust in terms of retention but sensitive in terms of quadrant classification.

Overall, the sensitivity analysis qualifies the interpretation of the original thematic map. The thematic structure is not entirely unstable, because sport and physical activity remain consistently positioned as motor themes. However, the classifications of climate change, environmental sustainability, and sustainability vary across specifications. Therefore, the revised interpretation avoids overclaiming fixed quadrant positions and instead treats these themes as threshold-sensitive elements of the evolving conceptual structure of sport sustainability research.

### 3.11 Thematic evolution across periods

The thematic evolution analysis was revised across three periods: 2010–2014, 2015–2019, and 2020–2025. Although early-access or metadata-indexed 2026 records appeared in the exported database files, these records were excluded from the thematic evolution analysis to avoid incomplete-year indexing bias. The periodization was defined on conceptual and historical grounds. The first period represents the pre-SDG foundational phase, the second period captures the post-2015 SDG institutionalization and diversification phase, and the third period represents the recent climate-accountability phase. The Bai–Perron breakpoint identified in the annual production analysis is interpreted as a production-level acceleration point rather than as a thematic boundary.

**Figure 12:**
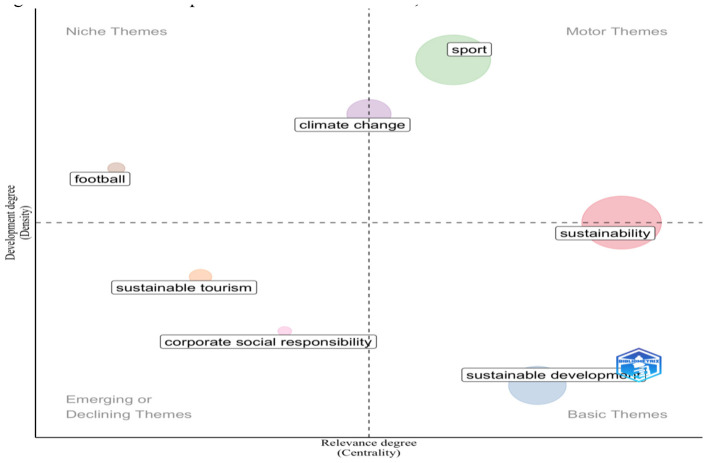
Thematic map of the foundational period, 2010–2014.

Figure 12 presents the thematic structure of the foundational period between 2010 and 2014. This period appears to be characterized by early sustainability awareness, sport event legacy, tourism-related sustainability, environmental impact, and sport participation. The thematic structure suggests that the early development of the field was closely connected to mega-events, tourism, and the long-term social and environmental consequences of sport-related activities.

During this period, sport sustainability research was still emerging as a distinct area of inquiry. The themes were likely more descriptive and context-specific, focusing on the environmental and social outcomes of sport events rather than on comprehensive sustainability governance. Therefore, Figure 12 indicates that the foundational phase was primarily concerned with recognizing sustainability as a relevant issue within sport settings.

**Figure 13:**
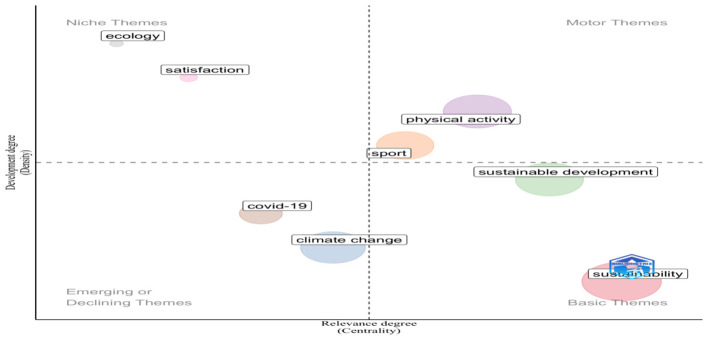
Thematic map of the diversification period, 2015–2019.

Figure 13 presents the thematic structure of the diversification period between 2015 and 2019. Compared with the foundational period, this phase appears to include a broader range of themes related to governance, sport management, sustainable tourism, institutional responsibility, social sustainability, and sustainable development. This diversification coincides with the increasing global visibility of the Sustainable Development Goals and the growing expectation that sport organizations contribute to sustainability agendas.

The thematic expansion shown in Figure 13 indicates that sport sustainability research moved beyond event-based environmental concerns and began to incorporate organizational, policy-oriented, and social dimensions. This period can therefore be interpreted as a stage of institutional broadening, in which sustainability became linked not only to environmental impact but also to management practices, stakeholder engagement, and social responsibility.

**Figure 14:**
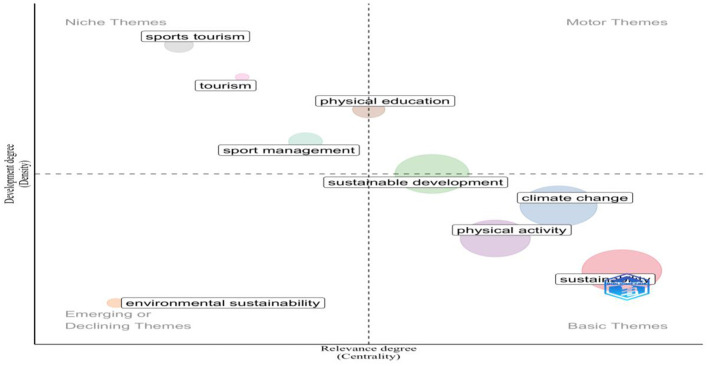
Thematic map of the recent climate-accountability period, 2020–2025.

Figure 14 presents the thematic structure of the recent climate-accountability period between 2020 and 2025. This period appears to be marked by stronger emphasis on climate change, sustainability, sustainable development, sport, football, sustainable tourism, and corporate social responsibility. Compared with earlier periods, the recent phase reflects a shift from broad sustainability awareness and institutionalization toward more policy-relevant and accountability-oriented research themes.

The position of “climate change” in the upper part of the map indicates that climate-related research has become internally developed, although its integration with the broader conceptual structure of the field remains partial. “Sport” appears as a motor theme, suggesting that sport-related research continues to function as a central and well-developed organizing theme. “Sustainability” and “sustainable development” remain central reference points, indicating that the recent period is shaped by both broad sustainability discourse and more operational concerns related to governance, responsibility, and accountability.

This interpretation should be understood as a bibliometric thematic tendency rather than direct evidence of organizational transformation. Excluding incomplete 2026 records reduces the risk of treating partial-year indexing effects as genuine thematic change.

### 3.12 Three-fields analysis: country–keyword–source relationships

**Figure 15:**
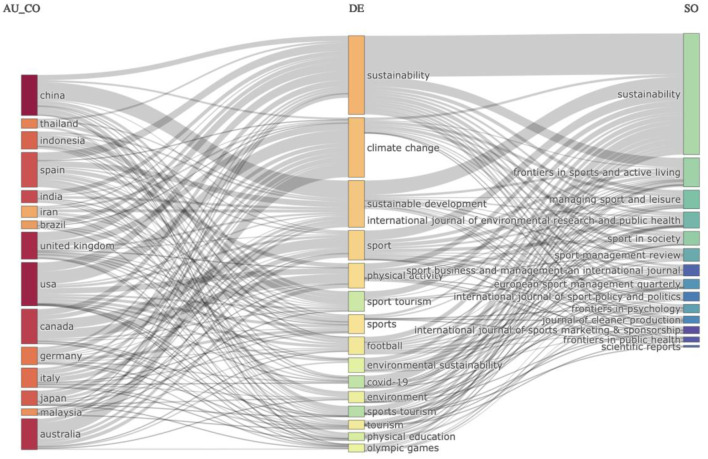
Three-fields plot of country–keyword–source relationships.

Figure 15 presents the three-fields plot linking countries, keywords, and publication sources. This visualization provides an integrated perspective on how geographical production centers are connected to dominant research themes and preferred publication outlets. Unlike isolated productivity indicators, the three-fields plot reveals the relational structure of the field by connecting actors, concepts, and journals within the same analytical frame.

The country–keyword relationships in Figure 15 suggest that leading countries are associated with central themes such as sustainability, climate change, sport management, tourism, sustainable development, and environmental management. This indicates that national research profiles are not defined only by publication volume but also by thematic specialization. Countries with strong publication output appear to contribute to the conceptual direction of the field by repeatedly engaging with key sustainability themes.

The keyword–source relationships also reveal the publication ecology of the field. Broad sustainability and environmental journals appear to be associated with themes such as sustainability, climate change, and sustainable development, while sport-focused journals are more closely linked to sport management, policy, tourism, and organizational themes. This structure confirms that sport sustainability research operates through a multi-channel publication system.

Overall, Figure 15 demonstrates that sport sustainability research has a relational, multi-layered knowledge structure. Countries, keywords, and sources do not operate independently; rather, they form an integrated intellectual system. This finding supports the interpretation that the field is shaped by the interaction of geographical research capacity, thematic specialization, and source-level publication structures.

## 4 Discussion

The findings of this study provide a multi-layered interpretation of sport sustainability research as an expanding, database-sensitive, and partially consolidated scientific field. Rather than treating bibliometric results as isolated indicators of publication volume, this discussion interprets them through the combined lenses of field formation, database-mediated visibility, scientific capital, thematic evolution, methodological robustness, and institutional sustainability governance. This approach is important because bibliometric structures do not merely describe how much research has been produced; they also reveal how scientific authority, conceptual boundaries, publication hierarchies, and research priorities are organized (Donthu et al., 2021; Zupic and Cater, 2015). In line with the Leiden Manifesto, bibliometric indicators are therefore interpreted contextually rather than as neutral proxies for scientific quality or field maturity (Hicks et al., 2015).

The revised findings suggest that sport sustainability research has moved from a fragmented collection of environmentally oriented studies toward a more recognizable knowledge domain organized around sustainability, sport, physical activity, climate accountability, ESG governance, sport ecology, and SDG-oriented research. However, the field should not be interpreted as fully consolidated. Its structure is marked by a tension between expansion and concentration: publication output and thematic diversity have increased substantially, but scientific capital remains unevenly distributed across authors, sources, databases, and thematic clusters. Interpreted through Bourdieu's sociology of science, this pattern suggests that sport sustainability research is becoming an emerging scientific field in which visibility, recognition, and authority are structured through publication, citation, journal positioning, institutional affiliation, and recurrent participation (Bourdieu, 1975, 1988, 2004).

This interpretation also extends previous bibliometric work on sustainability perspectives in sport. (Boros et al. 2024) provided an important antecedent by demonstrating the increasing visibility of sustainability perspectives within sport research. The present study builds on this foundation by using a larger dual-database corpus, explicitly comparing Web of Science and Scopus, quantifying database overlap, examining database-specific records, testing search-field sensitivity, validating deduplication reliability, and interpreting the results through an operationalized field-theoretical framework.

### 4.1 Sport sustainability as a partially consolidated scientific field

Sport sustainability research has undergone a structural transformation that this study maps through a comparative Web of Science–Scopus bibliometric design. The analysis of 6,279 unique publications reveals not merely growth in publication volume, but a reconfiguration of the field's intellectual architecture: from environmental awareness toward climate accountability, from event legacy toward ESG and SDG-oriented governance, and from disciplinary dispersion toward interdisciplinary institutionalization. This transformation is consistent with the broader development of sport sustainability and sport ecology as increasingly visible research areas within sport management and environmental scholarship (McCullough et al., 2020; Orr et al., 2022).

This transformation supports the interpretation of sport sustainability as an emerging scientific field. The field now contains recognizable publication centers, leading authors, international collaboration patterns, and identifiable thematic clusters. However, these features indicate partial rather than complete consolidation. Growth alone should not be equated with maturity. A field may expand rapidly while still lacking a stable author community, consistent thematic boundaries, and fully institutionalized research traditions. This interpretation is compatible with Bourdieu's view of scientific fields as structured spaces of competition, recognition, and symbolic capital accumulation rather than neutral collections of publications (Bourdieu, 1975, 1988, 2004).

The Lotka findings are particularly important for this interpretation. The original model showed a strong Lotka-like power-law structure, but the significant K-S deviation required deeper interpretation. The residual analysis clarifies that this deviation was mainly driven by an excess of single-publication authors. The observed number of single-publication authors was 16,498, compared with an expected value of 8,266, producing a large positive residual. This indicates that sport sustainability research attracts broad interdisciplinary participation, but much of this participation remains episodic rather than sustained.

This pattern suggests that the field has not yet developed a broad and stable mid-level author community. In Bourdieusian terms, scientific capital is beginning to accumulate, but it is concentrated among a relatively small recurrent author core rather than being widely distributed across the field (Bourdieu, 1975, 2004). Sport sustainability research is therefore best understood as a rapidly expanding but only partially consolidated scientific field.

### 4.2 Database infrastructures as boundary-setting mechanisms

The comparative Web of Science–Scopus design demonstrates that bibliographic databases do not merely store scientific records; they actively shape the visible boundaries of a field. This is a crucial methodological and epistemological point. Bibliometric maps should not be interpreted as neutral mirrors of knowledge. They are structured representations produced through database coverage, indexing policies, metadata architecture, language preferences, and journal selection practices (Hicks et al., 2015; Mongeon and Paul-Hus, 2016; Singh et al., 2021).

The database-specific analysis provides direct evidence for this argument. Shared records constituted the largest segment of the final dataset, accounting for 54.71% of all publications. These shared records also showed the highest citation visibility, suggesting that publications indexed in both databases represent the most institutionally visible bibliographic core of sport sustainability research. By contrast, WoS-only and Scopus-only records accounted for 20.10% and 25.20% of the final corpus, respectively, indicating that a substantial part of the field depends on database-specific coverage.

This finding qualifies the interpretation of the Jaccard coefficient. The overlap value should not be understood only as evidence of a common bibliographic core. It also reveals a substantial database-specific periphery. In other words, database choice affects not only the size of the corpus but also the apparent citation profile, source structure, temporal distribution, and thematic orientation of sport sustainability research. This supports previous arguments that major bibliographic databases provide overlapping but non-identical representations of scientific fields (Mongeon and Paul-Hus, 2016; Singh et al., 2021).

The comparison also shows why a dual-database design is necessary for an interdisciplinary field such as sport sustainability. A Web of Science-only design would have captured a more recent and more selective segment of the field, whereas a Scopus-only design would have captured a broader and somewhat older segment with distinct source-level visibility. Therefore, comparing Web of Science and Scopus is not only a technical improvement but also a way of reducing field-boundary bias in interdisciplinary bibliometric research. This is consistent with bibliometric guidelines emphasizing transparency in database selection, data processing, and interpretive limits (Donthu et al., 2021; Hicks et al., 2015; Zupic and Cater, 2015).

The search-field sensitivity analysis further reinforces this argument. The WoS Topic field includes title, abstract, author keywords, and Keywords Plus, whereas Scopus TITLE-ABS-KEY does not include an equivalent Keywords Plus field. The restricted WoS search excluding Keywords Plus retrieved fewer records than the full TS-based query, confirming that metadata architecture influences retrieval. However, the persistence of a large restricted-field corpus also indicates that the comparative findings are not merely an artifact of WoS metadata structure. Rather, the results show both a genuine database coverage difference and the need to interpret database comparisons with sensitivity to metadata architecture.

### 4.3 Scientific capital, author concentration, and publication hierarchies

The author and source-level findings also support the interpretation of partial consolidation. The presence of highly productive and highly cited authors indicates that a limited group of scholars has begun to accumulate scientific capital within sport sustainability research. However, this accumulation is uneven. Most contributors remain peripheral and episodic, while sustained visibility is concentrated among relatively few recurrent authors. This pattern is consistent with Bourdieu's argument that scientific authority is unevenly distributed within fields through accumulated recognition, publication visibility, and symbolic capital (Bourdieu, 1975, 1988, 2004).

The residual interpretation of Lotka's law strengthens this point. Authors with one publication accounted for 86.28% of all contributors, compared with an expected proportion of 43.23%. Authors with two and three publications also exceeded expected values, but the residuals became very small or slightly negative from four publications onward. This pattern indicates a weak transition from occasional contribution to sustained authorship. It also suggests that the field lacks a broad middle layer of recurring contributors who could provide long-term intellectual continuity.

Bradford's law further demonstrates that sport sustainability research has developed recognizable publication hierarchies. A limited number of core journals act as central publication spaces, while a much larger number of intermediate and peripheral journals support interdisciplinary diffusion. In Bourdieusian terms, Bradford core journals can be interpreted as consecrating spaces that help stabilize the field's symbolic order. Yet the large peripheral zone indicates that sport sustainability research continues to circulate across many adjacent domains rather than being contained within a narrow disciplinary center (Bourdieu, 1975, 2004).

This duality is characteristic of an interdisciplinary field in formation. Sport sustainability research has identifiable centers of authority, but it also remains open to diverse disciplinary inputs from sport management, sustainability science, environmental studies, public health, tourism, urban planning, and organizational research. This supports the argument that bibliometric maps are most useful when they are interpreted as contextual representations of field structure rather than simple rankings of productivity or impact (Donthu et al., 2021; Zupic and Cater, 2015).

### 4.4 Thematic consolidation and threshold-sensitive conceptual development

The thematic findings indicate that sport sustainability research has developed a recognizable conceptual structure, but the sensitivity analysis shows that this structure should not be interpreted as fully fixed. In the baseline thematic map, sustainability, sport, physical activity, sustainable development, tourism, environmental sustainability, and climate change formed the main thematic clusters. These results suggest that the field has a coherent conceptual architecture, but the robustness of each thematic position varies.

The sensitivity analysis is particularly important because keyword-based science mapping can be affected by term selection, threshold settings, classification decisions, and metadata structures (Waltman and van Eck, 2012; Zupic and Cater, 2015). Sport and physical activity remained consistently classified as motor themes across alternative threshold specifications, suggesting that they represent stable organizing concepts within the field. These themes appear to connect sustainability-related research to sport participation, sport management, and physical activity contexts.

By contrast, climate change, environmental sustainability, and sustainability showed threshold-sensitive quadrant positions. The classification of climate change as a niche theme in the baseline model should therefore be treated cautiously. Across alternative models, climate change shifted between niche and emerging/declining positions, indicating that it is better understood as a developing and structurally unstable theme rather than as a fixed specialized cluster. Environmental sustainability and sustainability also shifted across specifications, suggesting that their thematic roles are robust in terms of retention but sensitive in terms of quadrant classification.

This interpretation strengthens the methodological credibility of the study by acknowledging the limits of keyword-based thematic mapping. Thematic maps are useful for identifying conceptual organization, but their results are shaped by keyword frequency thresholds, term retention decisions, and metadata quality. Therefore, the revised interpretation treats the thematic structure as a structured approximation of the field rather than a definitive representation of its conceptual boundaries (Hicks et al., 2015; Waltman and van Eck, 2012).

### 4.5 From event legacy to climate accountability

The thematic evolution analysis suggests a broad transition from early sustainability awareness and event legacy toward more accountability-oriented research. The foundational period was more strongly associated with event impacts, tourism, participation, and environmental awareness. The diversification period reflected the influence of the post-2015 SDG agenda and the increasing incorporation of governance, management, and social responsibility themes. The recent climate-accountability period from 2020 to 2025 showed stronger emphasis on climate change, sustainability, sustainable development, sport, football, sustainable tourism, and corporate social responsibility.

This transition is consistent with sport ecology scholarship, which emphasizes the bidirectional relationship between sport and the natural environment and frames climate change as a structural condition affecting sport participation, facilities, scheduling, event operations, and athlete safety (McCullough et al., 2020; Orr et al., 2022). It also aligns with environmental sustainability research in sport, where organizational practices, stakeholder engagement, environmental management, and sustainability implementation have become increasingly important concerns (Cury et al., 2023).

This conceptual transition can also be interpreted through sustainability transitions literature. Sustainability transitions research emphasizes that systemic change involves shifts in institutions, practices, technologies, and governance arrangements rather than isolated organizational actions (Geels, 2011; Markard et al., 2012). From this perspective, the growing visibility of climate accountability, ESG governance, carbon-related concerns, and sustainability performance in sport research reflects a broader movement from sustainability as a symbolic or normative discourse toward sustainability as an operational, measurable, and governance-oriented challenge.

However, these findings should be interpreted as bibliometric evidence of changing research attention rather than direct evidence of organizational transformation. The increasing visibility of climate accountability, ESG governance, and sustainability performance indicates that the academic field is moving toward more measurable and policy-relevant research questions. It does not, by itself, prove that sport organizations have adopted such practices. Future empirical research is therefore needed to examine whether and how these scholarly themes are translated into organizational reporting, climate action, and sustainability governance.

The exclusion of incomplete 2026 records from thematic evolution analysis strengthens this interpretation. It reduces the risk of treating partial-year indexing effects as genuine thematic change and allows the recent period to be interpreted more cautiously. In this respect, the revised periodization provides a more reliable account of conceptual transition from sustainability as a normative ideal toward sustainability as a measurable organizational and policy challenge.

### 4.6 SDG diffusion and institutional governance in sport

The alignment between sport sustainability research and the SDGs should not be interpreted as a simple thematic coincidence. The SDGs function as a global goal-setting architecture that shapes governance priorities, institutional strategies, research agendas, and reporting practices. In the sport context, SDG-related themes are particularly relevant because sport is connected to health and well-being, sustainable cities, responsible consumption, climate action, and cross-sectoral partnerships.

The findings indicate that sport sustainability research is closely connected to SDG 3, SDG 11, SDG 12, SDG 13, and SDG 17. This pattern is conceptually coherent. SDG 3 is reflected in the visibility of physical activity, health, participation, and well-being. SDG 11 is linked to stadiums, sport infrastructure, mega-events, tourism, mobility, urban sport spaces, and community development. SDG 12 is reflected in green management, circular economy, responsible consumption, waste reduction, and sustainable facilities. SDG 13 is visible through climate change, carbon footprint, decarbonization, net zero, resilience, and sport ecology. SDG 17 is reflected in international collaboration patterns and the cross-sectoral nature of sport sustainability research.

(Campillo-Sánchez et al. 2021) argue that sport can contribute to the SDGs when policy coherence, institutional alignment, shared measurement frameworks, and multi-stakeholder cooperation are strengthened. The present study supports this argument but also qualifies it. The growing presence of SDG-related terminology in the literature does not automatically mean that sport organizations are producing measurable SDG outcomes. It may reflect genuine institutional transformation, but it may also reflect symbolic adoption, strategic communication, or isomorphic pressure within the global sustainability field.

From a sport governance perspective, this distinction is important. Governance in sport requires coordination among organizations, stakeholders, and public authorities, particularly when sport is connected to broader policy goals such as sustainability and accountability (Chappelet and Kübler-Mabbott, 2008). If sport organizations adopt SDG language without measurable indicators, transparent reporting, or verifiable outcomes, sport sustainability may become performative rather than transformative. Therefore, future research should examine whether SDG alignment in sport is supported by empirical evidence, including sustainability reports, carbon inventories, facility audits, stakeholder engagement data, and measurable social and environmental outcomes.

### 4.7 Implications for research, policy, and practice

The findings have implications for research, policy, and sport organizational practice, but these implications should remain within the evidentiary limits of bibliometric analysis. The study identifies the knowledge areas that are becoming increasingly visible in the literature, including climate accountability, ESG governance, sport ecology, sustainable development, environmental sustainability, and sustainability performance. These areas provide a research agenda for scholars interested in examining how sustainability is conceptualized, measured, governed, and reported in sport contexts.

For researchers, the study demonstrates the need to move beyond descriptive mapping toward theory-driven, database-sensitive, and measurement-oriented inquiry. Future bibliometric studies should report database overlap metrics, search-field sensitivity, deduplication reliability, thematic mapping robustness checks, and reproducibility materials. Such practices would improve transparency and reduce the risk of overinterpreting database-dependent patterns as objective field structures (Donthu et al., 2021; Hicks et al., 2015; Zupic and Cater, 2015).

Substantively, future empirical research should examine whether and how sport organizations translate sustainability discourse into measurable indicators, reporting systems, climate strategies, ESG practices, and governance mechanisms. This is especially important because bibliometric visibility does not necessarily imply implementation. A theme may become central in the literature before it becomes institutionalized in organizational practice. This distinction is consistent with prior work emphasizing that sport's contribution to sustainable development requires policy coherence, institutional alignment, shared measurement frameworks, and multi-stakeholder collaboration (Campillo-Sánchez et al., 2021).

Recent systematic evidence also suggests that sustainable development practices in professional sport organizations are becoming a distinct area of inquiry (Banucha et al., 2025). However, such work also reinforces the need to examine how sustainability is operationalized in organizational settings rather than simply assumed from the presence of sustainability language. Future studies should therefore connect bibliometric trends with evidence on organizational practice, including reporting systems, ESG indicators, carbon accounting, facility management, stakeholder accountability, and SDG alignment.

For sport governance, the findings suggest that future studies should move beyond documenting the presence of sustainability discourse and investigate measurable sustainability practices. Such research would help connect bibliometric trends with actual organizational behavior and policy implementation. Overall, the study shows that sport sustainability research is at a critical stage of development. It has achieved sufficient volume, thematic diversity, publication concentration, and intellectual leadership to be recognized as an emerging scientific field. However, its future development will depend on whether it can consolidate theoretically while remaining interdisciplinary, expand globally while avoiding knowledge asymmetries, and move from sustainability discourse toward measurable climate and SDG accountability.

## 5 Conclusion

This study examined the intellectual, structural, and conceptual evolution of sport sustainability research through a comparative bibliometric analysis of Web of Science and Scopus. By integrating 6,279 unique publications into a single analytical corpus, the study provides a comprehensive and database-sensitive map of how sport sustainability research has developed at the intersection of climate action, SDGs, ESG-oriented governance, sport ecology, and sport management.

The findings show that sport sustainability research has moved beyond a peripheral environmental concern and has become an increasingly visible interdisciplinary research field. However, the field should be interpreted as partially consolidated rather than fully stabilized. The annual production trend demonstrates sustained growth, particularly after 2018 and 2020, but the author productivity structure indicates that broad participation coexists with a narrow recurrent author core.

A central contribution of the study lies in its comparative Web of Science–Scopus design. The results show that the two databases share an institutionally visible bibliographic core while also contributing substantial database-specific records to the final corpus. Shared records accounted for 54.71% of the final dataset, whereas WoS-only and Scopus-only records accounted for 20.10% and 25.20%, respectively. This finding demonstrates that database choice affects not only corpus size but also citation visibility, source structure, temporal distribution, and thematic orientation. These results reinforce the methodological importance of dual-database designs in interdisciplinary bibliometric research.

The study also shows that sport sustainability research is developing the structural features of an emerging scientific field. The Lotka analysis revealed a strong Lotka-like power-law authorship structure, but the residual inspection showed that the significant K-S deviation was mainly associated with an excess of single-publication authors and a weak transition toward sustained authorship. Bradford analysis demonstrated a core–periphery publication structure, showing that the field has developed identifiable publication centers while remaining widely distributed across adjacent domains.

The conceptual analyses further reveal that the field has evolved from early concerns with event legacy, sport tourism, and environmental awareness toward climate accountability, ESG governance, sport ecology, sustainable development, and SDG-oriented research. The thematic mapping sensitivity analysis showed that some concepts, such as sport and physical activity, are stable organizing themes, whereas climate change and environmental sustainability remain threshold-sensitive and still-developing themes. This finding highlights the need to interpret keyword-based maps cautiously and contextually.

From a theoretical perspective, the study positions sport sustainability research as an emerging scientific field shaped by scientific capital, publication hierarchies, database visibility, thematic consolidation, and external institutional pressures. This field-theoretical interpretation helps explain why certain authors, journals, countries, and themes become central, while others remain peripheral. It also highlights the need to interpret bibliometric maps not as neutral mirrors of knowledge but as representations of institutionalized scientific communication shaped by database architecture, journal gatekeeping, citation conventions, and language biases.

Overall, this study contributes methodologically by demonstrating the value of dual-database bibliometric mapping, search-field sensitivity analysis, deduplication reliability testing, database-specific record comparison, Lotka residual interpretation, and thematic mapping robustness checks. It contributes conceptually by operationalizing Bourdieu's field theory through bibliometric indicators and by interpreting sport sustainability research as an emerging but partially consolidated scientific field.

The findings also identify important directions for future research. Bibliometric evidence indicates that climate accountability, ESG governance, sport ecology, SDG alignment, and sustainability performance are becoming increasingly visible in the literature. However, this visibility should not be interpreted as direct evidence of organizational transformation. Future studies should connect bibliometric trends with empirical data on the sustainability performance of sport organizations, events, facilities, and governing bodies. In particular, further research should examine how sustainability indicators, carbon reporting, ESG practices, SDG accountability, and climate governance are implemented, measured, and verified in sport contexts.

Future research should also expand the database scope beyond Web of Science and Scopus, include non-English and regional publications, examine Global South perspectives, and incorporate policy documents, gray literature, books, and organizational sustainability reports where appropriate. In doing so, future studies can move the field beyond mapping scientific communication toward evaluating how sport contributes to real-world sustainability transformation.

## Data Availability

The original contributions presented in the study are included in the article/Supplementary material, further inquiries can be directed to the corresponding author.
