# Supplementary material for: Sport sustainability research at the intersection of climate action, SDGs, and sport management: a comparative bibliometric mapping of Web of Science and Scopus
Source: Front Res Metr Anal. 2026 Jul 3;11:1884197. doi: 10.3389/frma.2026.1884197 (PMC13376297; doi:10.3389/frma.2026.1884197)
Supplement: Supplementary file 1 [file Table_1.docx]

Supplementary Table S1. Search-Field Sensitivity Analysis

| **Search Variant** | **Database** | **Search Field** | **Records** | **Difference From Main WoS Query** | **Percentage Change** |
| --- | --- | --- | --- | --- | --- |
| Main WoS query | Web of Science | TS | 4675 | Reference | Reference |
| Restricted WoS query | Web of Science | TI/AB/AK | 3969 | -706 | -15.10% |
| Main Scopus query | Scopus | TITLE-ABS-KEY | 5036 | — | — |

Supplementary Table S2. Inter-Rater Reliability of Deduplication Validation

| **Indicator** | **Value** |
| --- | --- |
| Validation sample | 200 records |
| Observed agreement (Po) | 92.5% |
| Expected chance agreement (Pe) | 59.0% |
| Cohen’s kappa (κ) | 0.817 |
| Interpretation | Almost perfect agreement |

Supplementary Table S3. Thematic Cluster Metrics Based on Centrality and Density

| **Theme Cluster** | **Centrality** | **Density** | **Rank Centrality** | **Rank Density** | **Quadrant** |
| --- | --- | --- | --- | --- | --- |
| Sustainability | 0.113 | 3.538 | 1.000 | 0.333 | Basic Themes |
| Sport | 0.101 | 3.604 | 0.833 | 0.500 | Motor Themes |
| Physical activity | 0.085 | 4.151 | 0.667 | 0.833 | Motor Themes |
| Sustainable development | 0.085 | 2.914 | 0.500 | 0.167 | Basic Themes |
| Tourism | 0.078 | 2.877 | 0.333 | 0.000 | Emerging/Declining Themes |
| Environmental sustainability | 0.069 | 4.340 | 0.167 | 1.000 | Niche Themes |
| Climate change | 0.032 | 4.146 | 0.000 | 0.667 | Niche Themes |

Supplementary Table S4. Keyword Network Centrality Metrics

| **Keyword** | **Degree Centrality** | **Betweenness Centrality** | **Closeness Centrality** |
| --- | --- | --- | --- |
| Sustainability | 1900 | 0.1755 | 0.3989 |
| Climate change | 1031 | 0.0952 | 0.3749 |
| Sustainable development | 924 | 0.0822 | 0.3810 |
| Sport | 682 | 0.0369 | 0.3558 |
| Physical activity | 591 | 0.0357 | 0.3560 |
| Sports | 524 | 0.0340 | 0.3546 |
| Football | 409 | 0.0304 | 0.3567 |
| Tourism | 379 | 0.0207 | 0.3493 |
| Environment | 339 | 0.0154 | 0.3474 |
| Olympic Games | 326 | 0.0136 | 0.3370 |

Supplementary Table S5. Database Overlap and Jaccard Similarity Metrics

| **Metric** | **Value** |
| --- | --- |
| Web of Science (Raw) | 4675 |
| Scopus (Raw) | 5036 |
| Merged Unique Dataset | 6279 |
| Absolute Overlap (Both) | 3435 |
| Only Web of Science | 1262 |
| Only Scopus | 1582 |
| Jaccard Similarity Index | 0.5471 |
| Overlap Percentage (%) | 54.71 |

Supplementary Table S6. Observed–Expected Residual Analysis of Lotka’s Law

| Articles Written | Observed Authors | Expected Authors | Observed (%) | Expected (%) | Residual (%) |
| --- | --- | --- | --- | --- | --- |
| 1.00 | 16,498.00 | 8,266.00 | 86.28% | 43.23% | 43.05% |
| 2.00 | 1,728.00 | 1,195.00 | 9.04% | 6.25% | 2.79% |
| 3.00 | 474.00 | 385.00 | 2.48% | 2.02% | 0.46% |
| 4.00 | 176.00 | 173.00 | 0.92% | 0.9% | 0.02% |
| 5.00 | 82.00 | 93.00 | 0.43% | 0.48% | -0.06% |
| 6.00 | 55.00 | 56.00 | 0.29% | 0.29% | 0% |
| 7.00 | 29.00 | 36.00 | 0.15% | 0.19% | -0.04% |
| 8.00 | 24.00 | 25.00 | 0.13% | 0.13% | 0% |
| 9.00 | 15.00 | 18.00 | 0.08% | 0.09% | -0.02% |
| 10.00 | 11.00 | 13.00 | 0.06% | 0.07% | -0.01% |

**Supplementary Table S7. Sensitivity Analysis of Thematic Mapping Across Alternative Thresholds**

| **Core concept** | **Baseline minfreq=3, n=250** | **Model A minfreq=5, n=250** | **Model B minfreq=3, n=100** | **Model C minfreq=5, n=100** |
| --- | --- | --- | --- | --- |
| Climate change | Niche Themes | Emerging/Declining | Niche Themes | Emerging/Declining |
| Environmental sustainability | Niche Themes | Niche Themes | Motor Themes | Motor Themes |
| Sustainability | Basic Themes | Motor Themes | Motor Themes | Motor Themes |
| Sport | Motor Themes | Motor Themes | Motor Themes | Motor Themes |
| Physical activity | Motor Themes | Motor Themes | Motor Themes | Motor Themes |
